# Runtime translation of OCL-like statements on Simulink models: Expanding domains and optimising queries

**DOI:** 10.1007/s10270-021-00910-0

**Published:** 2021-08-23

**Authors:** Beatriz A. Sanchez, Athanasios Zolotas, Horacio Hoyos Rodriguez, Dimitris Kolovos, Richard F. Paige, Justin C. Cooper, Jason Hampson

**Affiliations:** 1 Department of Computer Science, University of York, York, UK; 2 Kinori Tech, Merida, Mexico; 3 Department of Computing and Software, McMaster University, Hamilton, Canada; 4 Rolls-Royce, Control Systems, Derby, UK

**Keywords:** Model driven engineering, Interoperability, Epsilon, MATLAB Simulink, Query optimisation, Eclipse Modelling Framework

## Abstract

Open-source model management frameworks such as OCL and ATL tend to focus on manipulating models built atop the Eclipse Modelling Framework (EMF), a de facto standard for domain specific modelling. MATLAB Simulink is a widely used proprietary modelling framework for dynamic systems that is built atop an entirely different technical stack to EMF. To leverage the facilities of open-source model management frameworks with Simulink models, these can be transformed into an EMF-compatible representation. Downsides of this approach include the synchronisation of the native Simulink model and its EMF representation as they evolve; the completeness of the EMF representation, and the transformation cost which can be crippling for large Simulink models. We propose an alternative approach to bridge Simulink models with open-source model management frameworks that uses an “on-the-fly” translation of model management constructs into MATLAB statements. Our approach does not require an EMF representation and can mitigate the cost of the upfront transformation on large models. To evaluate both approaches we measure the performance of a model validation process with Epsilon (a model management framework) on a sample of large Simulink models available on GitHub. Our previous results suggest that, with our approach, the total validation time can be reduced by up to 80%. In this paper, we expand our approach to support the management of Simulink requirements and dictionaries, and we improve the approach to perform queries on collections of model elements more efficiently. We demonstrate the use of the Simulink requirements and dictionaries with a case study and we evaluate the optimisations on collection queries with an experiment that compares the performance of a set of queries on models with different sizes. Our results suggest an improvement by up to 99% on some queries.

## Introduction

Systems engineers typically treat models as living entities, which must be modified and manipulated throughout the engineering lifecycle. In model-driven engineering processes specifically, models are transformed, queried, modified and validated (amongst other activities) with the aid of model management frameworks. In the case of such open-source frameworks like as QVT, ATL, Acceleo and Epsilon, these are mostly tailored for managing models conforming to the Eclipse Modelling Framework (EMF [42]), a de facto standard for domain-specific modelling [18]. Engineers working with modelling environments that build atop EMF, such as Papyrus [44], Capella [37] and SCADE [1], have at their disposal the model management facilities that these frameworks provide.

MATLAB Simulink is a modelling framework for dynamic systems that is widely used across many industries including aerospace and automotive [2, 35, 36]. This framework has its own set of model management activities to operate on its own models such as code generation and validation, but it does not offer facilities to export these models in XMI, the default exchange format for EMF models. As such, involving Simulink models in model management activities outside of MATLAB—particularly those involving other heterogeneous models—can be challenging.

There are multiple scenarios in which Simulink models are required to be used outside MATLAB. For example, the interface of its elements (inputs and outputs) could be parsed and stored in other models (e.g. XML) or used to produce documentation. Similarly, a number of works have been written on transformations that produce Simulink models from SysML models [3, 32, 39, 49]. In this particular scenario, several approaches achieve the transformation by generating MATLAB programs that produce the Simulink models on execution. Evidently, these approaches are less reusable for other scenarios, e.g. which may perform slightly different transformations, as they are written for a particular input model; but also because they only generate the model leaving out the possibility of reading it or modifying it. The Massif [48] project offers a more reusable approach that makes Simulink models available to model management frameworks with EMF support; this is achieved by transforming Simulink models into an EMF-compatible representation and vice versa. With this approach, the full Simulink model must be translated into EMF. This upfront transformation can be crippling for large models (as demonstrated in [38]) and unnecessary when the model management programs do not work on the entire model. Additionally, Simulink models that continuously evolve may require the co-evolution of the EMF-counterpart which involves the re-execution of a non-incremental transformation which can be expensive for large models. Furthermore, model management programs might be limited by the set of model element types supported by the Simulink-to-EMF transformation [31] which currently does not support Stateflow blocks.

Since Simulink is a tool that allows the creation of large and complex designs [30], we anticipated that the upfront transformation required with Massif would be expensive for these models. As such, we set out to implement an alternative approach that would shift the cost away from their EMF transformation and into the complexity of the manipulating program. Our approach consists in translating model management operations into small MATLAB programs at runtime (on-the-fly). This ensures a constant synchronisation between the modelling tools and the MATLAB models. Since no upfront transformation is required, the round-trip engineering and co-evolution costs are eliminated. Our implementation offers broader model coverage by including Stateflow elements, Simulink requirement and Simulink dictionary models. Additionally, it offers a more unified way of accessing model element properties and shares a vocabulary closer to the one used by MATLAB.

We compare the performance of our approach against Massif’s upfront model transformation by measuring the execution time of different stages of a representative model validation process. This process involves the execution of OCL-like invariants that validate structural properties on a sample of the largest available Simulink models on GitHub. Our evaluation indicates that our approach is more appropriate for continuously changing models as it can reduce the overall time of the validation process by up-to 80%. In contrast, the transformation-based approach (Massif) is better suited for signed-off models that need to be extensively queried as the cost of the transformation is a one-off and the validation two orders of magnitude faster.

Although experimental results on our on-the-fly Simulink bridge approach [38] show that it can reduce the overall execution time for a set of validation tasks on large models, the execution time was still high for certain classes of queries. We identified queries on collections of model elements as an area for optimisation. In order to improve the performance of our solution, we rewrite and delegate the execution of bulk queries to the MATLAB engine to take advantage of its inner indexes that are inaccessible by external clients. Experiments with models that grow exponentially in number of elements suggest that off-loading to MATLAB these queries can improve their performance by up to 99% in some cases.

Another area we identified as source of improvement was the coverage of the Simulink modelling environment which relies on a set of (different) MATLAB-based models. This is the case of Simulink requirements and Simulink dictionaries which add information to the Simulink models. In this paper, we expand our driver to support these additional model formats.

Our approach offers researchers and practitioners an additional option to manage Simulink models from model management frameworks that is convenient for large and/or continuously evolving Simulink models. Our implementation atop Epsilon, which offers a set of model management languages, makes this approach accessible to a range of model management activities such as model validation, model-to-model and model-to-text transformations, model comparison, etc., that can involve multiple heterogeneous models (e.g. EMF, UML) in the same program. Developers of model management frameworks such as ATL can use the technical details from this work to add support for Simulink models in their own frameworks.

As shown later in the paper, MATLAB uses a different set of operations and properties to manage elements in the different types of Simulink models (dictionaries, requirements, Simulink models) and even for managing different elements within these models, as is the case of Simulink and Stateflow model elements. A side contribution of our approach is that it provides a unified syntax to manage model elements within the same MATLAB model and across the different types of models.

This paper is an extended version of the work presented in [38]. Compared to [38], in this paper, we also:

- Propose and implement a driver with a similar approach to [38] that supports the management of Simulink dictionaries (Sect. 3.2) and requirements (Sect. 3.3). We also demonstrate how they can be used in a running example (Sect. 4).
- Propose a method for optimising queries on collections of Simulink and Stateflow model elements (Sect. 3.1.2).
- Evaluate the performance of proposed collection query optimisations with an experiment performed on model element collections of different sizes (Sect. 5.2). This experiment shows that the optimised queries outperform the original ones, some by up to 99%.
- Extend our review of related work section (Sect. 7).

*Roadmap* The rest of the paper is structured as follows. Section 2 introduces the modelling technologies used in our approach and evaluation. Section 3 presents the architecture of our “live” approach to bridge MATLAB Simulink models, requirements and dictionaries into Epsilon. In addition, this section presents a query optimisation approach which works on collections of Simulink and Stateflow model elements. Section 4 presents a running example that showcases potential usage of the drivers. Section 5 evaluates the performance of two approaches to bridge Simulink models: the upfront Simulink-EMF transformation against the on-the-fly MATLAB function execution. This section also evaluates the performance of query optimisations on collections of Simulink/Stateflow model elements. Section 6 discusses observations and lessons learned. Section 7 summarises related work. Section 8 concludes the paper and discusses future work.

## Background

In this section, we introduce the modelling technologies at the core of this work: MATLAB/Simulink, Epsilon, EMF and Massif.

### MATLAB/Simulink

MATLAB is a commercial tool developed by MathWorks that provides a variety of numerical computing environments. Under its Simulink [25] environment, it provides a graphical block-based modelling framework that supports the design, simulation and analysis of dynamic systems as well as model management activities like code generation and continuous model verification for such systems.

**Fig. 1:**
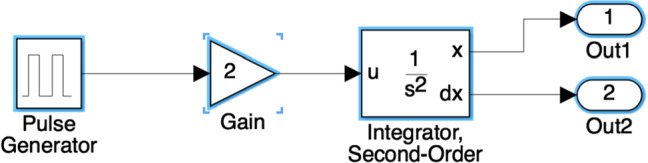
Example MATLAB/Simulink model

*Simulink Models* These are file-based models that represent dynamic systems based on interconnected blocks. A sample Simulink model representing the behaviour of a car in motion after the accelerator pedal [24] is presented in Fig. 1. The model contains five blocks from the Simulink library: a pulse generator, a gain, a second-order integrator and two outports. The pulse generator produces an input signal which simulates the accelerator pedal. The gain simulates the multiplied effect in the car acceleration. The second-order integrator enables the acquisition of the position and speed of the car from the acceleration through its outports. These blocks are interconnected by their ports through directed lines called signals.

Simulink model elements have both a type and a subtype. Example model element types include Block, Line and Port. Elements of *type* Port may have an inport or outport *subtype*. The list of subtypes is much longer for Block elements. All elements in Fig. 1 are blocks and their subtypes, from left to right, are: DiscretePulseGenerator, Gain, SecondOrderIntegrator and Outport.

**Fig. 2:**
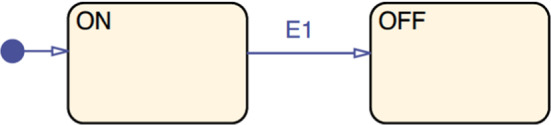
Example of MATLAB/Stateflow model elements

*Stateflow* MATLAB offers an additional toolbox of decision logic, called Stateflow [26], used to describe how blocks react to events, input signals and time-based conditions. This toolbox is based on state machines and flowcharts that can be attached to Simulink model elements. Figure 2 shows a sample Stateflow diagram containing two states named ON and OFF representing the operating modes of a system, and one transition,^1^ named E1, that connects one state to the other.

Stateflow model elements are persisted within a Simulink model. On a Simulink model, there is a corresponding Stateflow machine which contains all Stateflow charts of the model. Each chart defines decision logic by combining logical elements such as states, boxes, functions, data, events, messages, transitions, junctions and annotations. Only states, boxes and functions may contain any other logical elements indefinitely. Stateflow charts may be included as blocks in the Simulink model.

All model elements in Stateflow are Stateflow.Object instances and their specific type names are always preceded by the Stateflow prefix and a period. For example, states are of type Stateflow.State.

*Simulink functions* Simulink models can be manipulated manually using MATLAB’s graphical interface or programmatically invoking Simulink functions via MATLAB’s command line interface. Listing 1 illustrates some of the main Simulink functions that enable model navigation and modification.
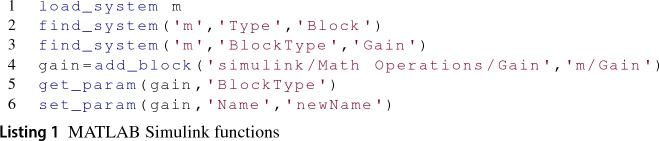


Line 1 shows how to load a model named m (same as its filename without extension) before we can interact with it. Line 2 shows how to retrieve all model elements of a given type, in this case, of elements of type Block from model m. For the model in Fig. 1, this evaluation would return five blocks. By changing the value of the type parameter to Line or Port (instead of Block) the same evaluation would return the 4 signals or 8 ports from the figure, respectively]. To find block model elements by their subtype it suffices to change the type keyword for BlockType in the find_system function. Line 3 illustrates query at subtype level which looks for block elements of subtype Gain. A similar approach applies for line and port elements which must replace the BlockType keyword for the corresponding LineType or PortType.

Line 4 illustrates the creation of a block of type Gain. The first function argument is the path of the library block to be used while the second argument represents the location in the destination model where the block will be created. This path starts with the name of the Simulink model, ends with the new element’s intended name, and may contain in-between the name of intermediary nested blocks that will contain the new element. Regarding the management of model element properties, line 5 gives an example of how to retrieve the subtype property of a gain block while line 6 shows how to set the block’s name.

*MATLAB Java API* MATLAB provides several Application Programming Interfaces (APIs) that allow the invocation of MATLAB functions from languages like Python, C, C++, Fortran and Java. In the case of its Java API, MATLAB provides the MatlabEngine class that is able to start or connect to a MATLAB engine and also to evaluate MATLAB functions. The Java API also provides wrappers for specific MATLAB types such as structural arrays, cell arrays, etc.

Listing 2 illustrates a sample program that starts a MATLAB engine (line 1), evaluates MATLAB functions (lines 2–3) and then closes the connection with the engine (line 5). The evaluation of MATLAB functions through the engine is done using the eval method which receives the functions as strings. Line 4 shows how the getVariable method can then be used on the engine to retrieve variables from MATLAB’s workspace. 
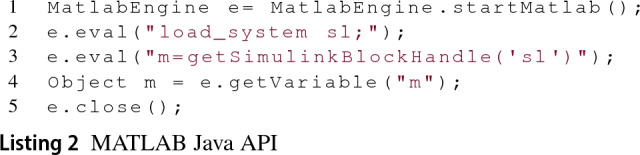


*Simulink Projects* MATLAB can group multiple Simulink models inside a project. Additionally, Simulink projects can contain requirement definitions, test cases and data dictionaries that complement the Simulink models.

A data dictionary file represents a data repository which includes design data such as parameters and signals that are used to configure the behaviour of Simulink models [28]. The dictionary object is the root element and it contains sections which in turn contain a set of entry elements. Each entry has a unique name and a value of an arbitrary type. The four default sections of a dictionary are named: Design Data, Configurations, EmbeddedCoder and Other Data.

The Simulink requirements toolbox enables the definition of requirements that can be linked to dictionary and Simulink model elements. The RequirementSet is the root object of a requirements definition file and contains elements of type Requirement, Justification and Reference. Each of these elements may contain nested elements of the same parent type, e.g. a requirement can contain other requirements. Elements of type Justification are requirements excluded from implementation and verification metrics, while Reference elements are proxies for external requirement objects from third-party applications.

Note that the traceability information—in the form of links among requirement, dictionary and Simulink model elements, is persisted in its own file. Each Simulink model, dictionary or requirement file which has elements involved in traceability links will have a corresponding link file with the same name as the model. At the root of element of these link files is the LinkSet which contains the set of Link elements.

### Epsilon

Epsilon is a framework of inter-operable languages and tools designed for model management tasks like model navigation, validation and transformation. The Epsilon Object Language (EOL) [19] is an OCL-like model query and transformation language that all other Epsilon languages are built on top of. Among these model management languages, we find the Epsilon Validation Language (EVL) [21]—designed to evaluate invariants on model elements, and the Epsilon Transformation Language (ETL) [20]—targeted at model-to-model transformations.

**Fig. 3:**
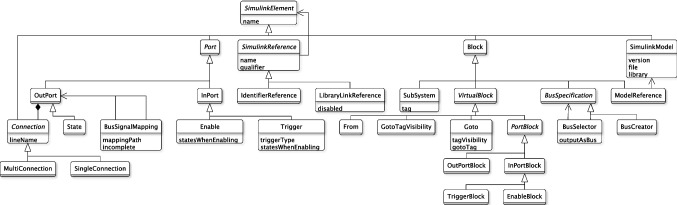
Epsilon architecture

Epsilon has a layered architecture (see Fig. 3). The Epsilon Model Connectivity (EMC) layer provides abstraction facilities that allow models of arbitrary technologies (e.g. EMF, XML) to be managed in a uniform manner in any of the Epsilon languages. Concrete EMC implementations for different modelling technologies such as EMF, or PTC Integrity Modeler, are known as (epsilon model) drivers.

Listing 3 shows a sample EOL program that navigates and manipulates a model M^2^ of arbitrary underlying modelling technology (e.g. EMF, XML). In the first line, the first of all the elements of type Block contained in the model is selected and then assigned to a new variable named element. In line 2, the value of its name property is retrieved, while in line 3, its evaluate() method is invoked. Further down, line 4 shows how a new element of type Block is created and assigned to the newElement variable while line 5 sets its name property.
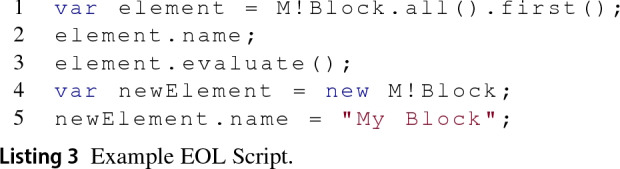


The EOL program in Listing 3 can be executed on models of arbitrary technology because the model is injected to the EOL interpreter at runtime by an arbitrary driver. The syntax that an EOL program uses to create and delete model elements, to set and get their properties, or invoke their methods does not depend on the driver. The contribution of a driver on any Epsilon program is the availability of model element types, their properties and additional methods at runtime. For example, the first() operator works on collections and is handled by the EOL engine by default.^3^ In contrast, the all() method in Listing 3 delegates the collection of all elements of type Block to the driver that handles model M. For Listing 3 to terminate successfully, the driver that provides and manages model M would need to know how to handle model elements of type Block with a name property and an evaluate() method.

Epsilon currently provides drivers for a variety of modelling technologies including EMF, XML [19] and Spreadsheets [12]. Section 3 presents the architecture of the Simulink driver which was the main contribution of the original publication [38], and of the Simulink requirements and Simulink dictionary drivers, which are introduced for the first time in this paper.

### The Eclipse Modelling Framework and Massif

The Eclipse Modelling Framework (EMF) was designed to build Java applications based on domain-specific model definitions [40] described with the Ecore meta-modelling language. EMF offers several representations for their models including Java code, XML Schema and UML diagrams, but its canonical format is the XML Metadata Interchange (XMI). Models conforming to an Ecore metamodel are referred to as EMF models.

**Fig. 4:**
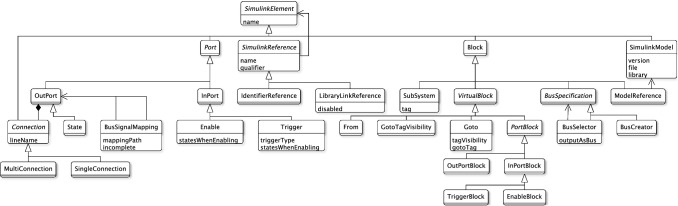
Simulink element types provided by Massif’s Simulink meta-model

*Massif* The Massif [48] project enables the transformation of MATLAB/Simulink models into an EMF-compatible representation and vice versa. Massif connects to MATLAB’s engine to parse and update Simulink models. The resulting EMF models conform to an Ecore Simulink meta-model defined by Massif which is limited to Simulink elements, i.e. leaving out Stateflow elements.

*Massif’s Simulink Ecore meta-model* The Massif meta-model considers any Simulink model element that can be identified and named as a subtype of the SimulinkElement class. All subclasses of SimulinkElement are presented in Fig. 4. Its direct descendants are Connection, Port, Block and SimulinkModel.

The SimulinkModel class is the root model element which keeps a reference to the file and version of the Simulink model. This class contains all the Block elements along with their Port and Connection elements.

In Massif, the ports (Port) of a block are either of type InPort or OutPort and they can be represented by a virtual block of class PortBlock. Similarly, the lines that connect the block ports are instances of the Connection class which can be either SingleConnection or MultiConnection. Any block whose MATLAB subtype cannot be found as a class in Massif is considered as a generic Block. Some blocks have predefined properties as attributes, e.g. the tag property in the SubSystem class but most of their properties are dynamically added to their parameters attribute which contains array of Property elements, each with a name, value and type.

Some of the Massif meta-model constructs differ from the way MATLAB manages Simulink models. The most notable difference is that Simulink’s block library offers 140 different Block subtypes (e.g. Gain, Sum, UnitDelay, etc.), while Massif only provides 11 concrete ones. The Simulink subtype of blocks that do not fall under the previous 11 subtypes can be retrieved from the block’s parameters attribute, looking for the one with the BlockType identifier. Similarly, there are 5 Port subclasses in Massif’s meta-model out of the 6 subtypes found in the Simulink library and, in particular, it is unclear how the State class in Massif maps to one or both of the Reset and ifaction port types in Simulink. A related inconsistency can occur when, after a transformation into EMF, the attributes of some block subclasses can have redundant or unpopulated values as they can also be found within the block’s parameters attribute, e.g. the tag attribute in the SubSystem class which can also be found in the parameters. Another difference is that the Connection class in Massif refers to Simulink model elements of type Line and subtype signal and that the MultiConnection and SingleConnection subclasses in the meta-model are used to refer to the SegmentType property of lines in MATLAB which can take the value of trunk or branch, correspondingly. In addition, subtype capitalisation is important for Simulink functions, e.g. input is used to refer to a port subtype as opposed to Input which identifies a block subtype. By contrast, in Massif the InPort and InPortBlock classes are used to refer to the port and block elements, respectively. Finally, MATLAB also handles special data types such as Cell Arrays^4^ and Structure Arrays^5^ which Massif stores as plain Strings.

*From Simulink to EMF and vice versa* Massif provides four different ways to transform Simulink models into an EMF-compatible representation. This process is known as the *import* process. The import modes can affect performance of the process as they differ in the way the MATLAB/Simulink ModelReference blocks^6^ are resolved: The *shallow* mode does not process the referenced model; the *deep* mode creates new SimulinkModel elements for each ModelReference block; the *flattening* model processes these blocks as SubSystem blocks; and the *referencing* mode processes ModelReference blocks as new EMF resources (once) and references them in the model.

The Massif *export* process transforms the Simulink EMF-compatible representation into a Simulink file. This process can produce files with either .slx or .mdl extension.

## Live Simulink bridges

In this section, we introduce the architecture and implementation of an approach that bridges models of the MATLAB Simulink environment with the Epsilon model management framework through on-the-fly translations of model management constructs into MATLAB functions. We choose the Epsilon [46] model management framework to implement and evaluate our approach based on the connectivity facilities that it offers and for the variety of model management languages in which the implementation becomes available. A similar approach can be implemented by other model management frameworks with similar connectivity facilities, such as ATL [17].

We present three concrete implementations (known as drivers or EMCs) that bridge different Simulink-based models with Epsilon. The Simulink EMC (Sect. 3.1) manages Simulink models including their Stateflow model elements. The Dictionary EMC (Sect. 3.2) handles Simulink dictionaries that are used by Simulink models to configure their models. The Requirements EMC (Sect. 3.3) can manage requirements that are linked to elements on dictionaries and Simulink models. The Simulink driver was formerly presented in [38], while the other two drivers are introduced for the first time in this work. All drivers are publicly available as plugins of the Epsilon project [46].

*Implementation* The Epsilon Model Connectivity (EMC) layer enables the uniform navigation and manipulation of models in any Epsilon model management language regardless of the model’s underlying technology. Each driver implementation is able to access and interact with “live” Simulink models as they generate on-demand MATLAB commands that are executed on the Simulink model. To achieve this, these drivers connect to MATLAB’s engine via the MATLAB Java API.

To illustrate the on-the-fly translation from EOL to MATLAB functions, consider the EOL program in Listing 4. At runtime, this program receives a model managed by the Simulink EMC driver, which can handle elements of type Block and knows how to manipulate their properties. The EOL Block.all() statement is used to retrieve all the Simulink block model elements from the model. To collect these elements the Simulink driver replaces the ? placeholder in the MATLAB function from line 1 in Listing 5 with the appropriate values, in this case the name of the model and the kind of element looked for, i.e. Block. The resulting function (line 2) is then submitted for evaluation to the MATLAB engine through its Java API. The function returns a collection of block identifiers which is wrapped by the Simulink EMC into a lazy collection of SimulinkBlock instances to be used in subsequent processing. The first() statement from our EOL program in Listing 4 is then called on this lazy collection of SimulinkBlock elements. This statement is an Epsilon operation that works on collections of any type to return their first element. The following statement Name is acting on the first SimulinkBlock returned. Since this model element belongs to the Simulink model handled by the Simulink EMC driver, it is the driver which handles the requested property access. To do so, the driver replaces the ? placeholder in line 3 of Listing 5 and submits its populated version (line 4) to the MATLAB engine over the API. The get_param MATLAB function in this place is expecting as first argument the block’s identifier (or handle) which is a number of type double. The last step consists in parsing the function result and assigning its value to the EOL variable name. 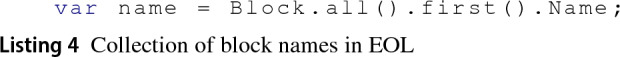

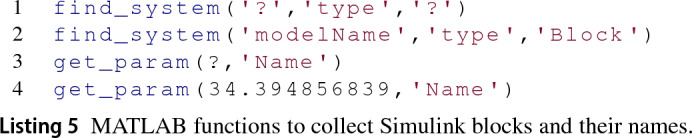


**Fig. 5:**
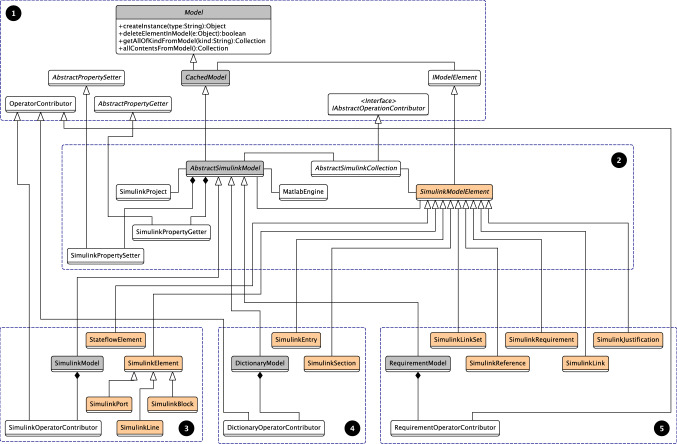
Simplified architecture of Simulink drivers. Group 1 represents the Epsilon Model Connectivity (EMC) Layer. Group 2 contains the Common Simulink EMC facilities. Groups 3–5 show the main contents of the Simulink model, Simulink dictionary and Simulink Requirement EMCs, respectively

*Architecture* Figure 5 shows the architecture of Simulink-based drivers and how they relate to the core facilities of the Epsilon Model Connectivity layer (Group 1). All concrete drivers such as the Simulink EMC (Group 3), the Dictionary EMC (Group 4) and the Requirements EMC (Group 5) use common classes and helpers that are provided by the abstract Common Simulink EMC (Group 2) which extends the core EMC. The common facilities include the configuration of the Simulink project and the establishment of a connection with the MATLAB engine. In addition, a set of abstract classes to handle lazy collections of Simulink-based model elements are also provided. Each concrete driver extends the AbstractSimulinkModel class and implements its own approach to create, delete and collect elements of specific types. This is done by overriding the respective methods from the Model superclass.

### Simulink EMC

This driver manages Simulink and Stateflow model elements which are described in Sect. 3.1.1. We expand on previous work by adding query optimisations on operations that act on collections of model elements which are presented in Sect. 3.1.2. A simplified view of this driver’s architecture is presented in Group 3 of Fig. 5.

#### Simulink

*Model* The Simulink EMC driver considers a Simulink file (*.slx or *.mdl) as a model. This model is managed as an instance of the SimulinkModel class (see Fig. 5). A model defines the behaviour of inherited methods from the class AbstractSimulinkModel in the Common Simulink EMC layer which in turn extends functionality from the CachedModel class defined in the EMC layer. Together, these classes describe how a model will perform CRUD operations on its *owned* model elements and the model itself, while they also determine how to *load* and *dispose* the model instance before and after the execution of a model management program, e.g. validation, transformation.

*Model Elements* The SimulinkModel manages elements that inherit from the SimulinkModelElement class which can be either SimulinkElement or StateflowBlock. For each MATLAB Simulink type, e.g. Block, Port and Line, there is a corresponding class, e.g. SimulinkBlock, SimulinkPort and SimulinkLine that extends SimulinkElement. These classes provide additional methods, e.g. to link blocks or to change their parents; and may override the behaviour of CRUD operations for the type of element they work on.

As discussed in Sect. 2, Simulink elements in MATLAB have subtypes, e.g. an element of type Block may be of subtype Gain or SubSystem. In Epsilon, the union of an element’s super types and of its concrete type is referred to as the element’s *kinds*. The Simulink EMC driver considers the Simulink subtype (e.g. Gain) as the model element concrete type, while still considering their Simulink type (e.g. Block) as one of their kinds. Stateflow element types (e.g. Stateflow.State) are used as their concrete type in Epsilon. At the same time, all Stateflow elements belong to the Stateflow kind in Epsilon.

MATLAB Simulink model elements provide different ways to be identified (e.g. *path*, *id*, *handle*). However, MATLAB Simulink functions return either *handles* or *paths*. As such, for Simulink elements, the driver uses as identifier their *handle* property which is a non-persistent session-based immutable identifier of type Double. In contrast, the driver uses the *id* property (Integer) to manipulate Stateflow elements which is easy to retrieve from the Stateflow objects returned by most Stateflow functions and queries. In the rest of the paper, we use interchangeably the words *identifier* or *handle* of a model element to refer to the mechanism by which specific element instances are retrieved across MATLAB toolboxes.

*Create element* The SimulinkModel instance manages the creation of Simulink and Stateflow model elements. When the reserved word new precedes a type name in an Epsilon program, the interpreted invokes the method createInstance(type:String) of the EMC model.

To instantiate Simulink blocks, MATLAB requires the path of the block in the Simulink library. The user is responsible for providing this path in order to instantiate a block in Epsilon. Once provided, the model populates the MATLAB function add_block with the path of the library block then asks the MATLAB engine to evaluate it. Listing 6 shows the creation of Sum and SubSystem blocks in EOL using their library block path.^7^ The Simulink driver creates these blocks at the top level of the Simulink model but they can later be placed elsewhere by changing their parent. 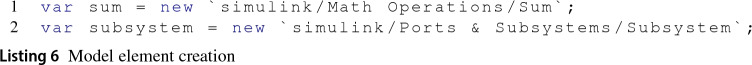


There is no equivalent add_port function in MATLAB to create port model elements. In contrast, the add_line MATLAB function which creates lines, requires the source and target ports to be connected. The Simulink EMC driver does not allow the direct creation of lines through EOL statement such as  or . Instead, lines are created using *linkage* methods on block elements which may specify the source and/or target ports to be connected. For example, provided the model from Fig. 1 with no lines, these can be created with the following EOL program: 
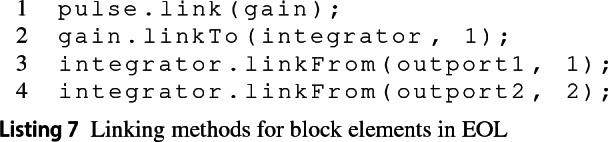


In MATLAB, Stateflow elements use a different syntax for instantiation which consists of their type followed by a container. For example, a Stateflow state can be created by invoking the function in Listing 8 where chart is the container Stateflow element. This same statement can be used in EOL to instantiate this state by preceding it with the new reserved word (line 1). Additionally, the Simulink EMC can delay the instantiation of Stateflow elements until the parent is assigned. In other words, a placeholder is created when using a statement with no parent (line 2) which is only submitted to the MATLAB engine for instantiation when its parent property is assigned (line 3). Before then, other properties of the Stateflow element can be assigned in memory to its placeholder. These properties are submitted to MATLAB just after the element is instantiated. 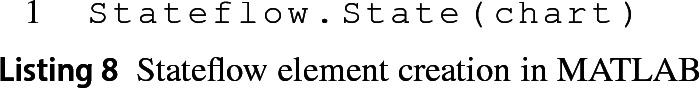

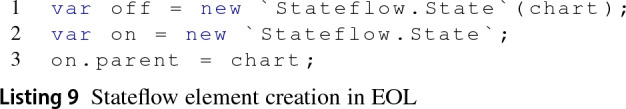


*Delete element* In Epsilon programs, deleting a model element involves the use of the delete reserved word before the element to delete as shown in Listing 10. 
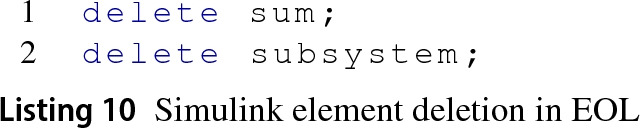


functions from lines 1 and 2 in Listing 11, respectively. There is no equivalent delete_port MATLAB function. 
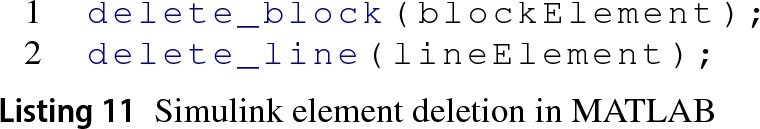


The SimulinkModel is responsible for the deletion of model elements and does this through its deleteElementInModel(e:Object) EMC method . For Simulink elements, the Simulink EMC chooses the appropriate MATLAB function for the element being deleted and provides its appropriate identifier. MATLAB has a different syntax to delete Stateflow elements which is the dot notation, e.g. elementId.delete.

*Read and update element properties* The SimulinkModel delegates to instances of the SimulinkPropertyGetter and SimulinkPropertySetter classes the responsibility of reading and updating properties of model elements. The former receives a model element and the property that is to be retrieved from it while the latter additionally requires the value to be assigned to the element’s property.

Depending on the kind of model element that these act upon, they populate and evaluate different MATLAB functions. For example, when dealing with Simulink blocks, these property *managers* evaluate the MATLAB functions from Listing 12. 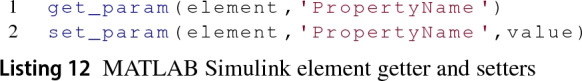


An example of an EOL program retrieving and populating Simulink element properties is shown in Listing 13. 
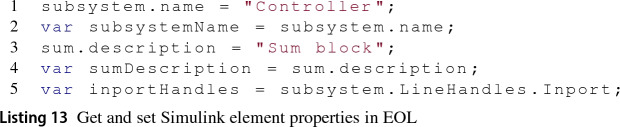


Lines 1 and 3 set element properties, while lines 2, 4 and 5 get property values from them. In the particular case of line 5, the property LineHandles returns a Structured Array, which is a MATLAB-specific type that represents an array of key-value pairs. In MATLAB, their values are retrieved using the getfield(element,property) function. The Simulink EMC driver can identify these types and navigate them as any other property. In the example, the value of its Inport key is retrieved.

In MATLAB, the dot notation is used once more to get and set properties from Stateflow elements. This is illustrated in Listing 14 where the name of a Stateflow State element is retrieved (line 1) and set (line 2). The syntax to do the same in an EOL program would be identical. 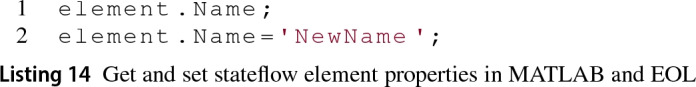


*Retrieve elements* To collect all instances of a given type, Epsilon programs use the all() operation on types. Alternatively, to collect all available elements on the model, Epsilon provides the allContents() operation at the EMC model level. Given a model M, Listing .15 illustrates different ways to retrieve Simulink model elements in EOL. 
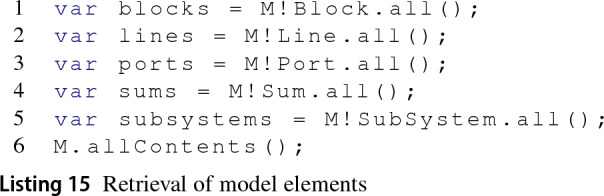


The all() operation (lines 1–3) triggers the execution of the getAllOfKindFromModel(kind:String) method from the SimulinkModel. At first, this method attempts to find elements of either Block, Line, Port or Stateflow kind. If the kind argument does not match any of those, as in lines 4–5, then the SimulinkModel will attempt to find the MATLAB subtype, e.g. SubSystem blocks or Stateflow.State elements. In contrast, the use of the allContents() in line 6 triggers the result aggregation of collections by kind, i.e. Block, Port, Line, and Stateflow elements.

Line 1 in Listing 16 reminds the reader how elements of type Port, Block or Line can be collected in MATLAB, while line 2 shows how this function is adapted to collect elements by their subtype. The SimulinkModel class populates and submits the appropriate MATLAB functions for the element kinds (e.g. Block) or types (e.g. Sum) to be collected and then stores the results in lazy collection objects which extend the AbstractSimulinkCollection class. 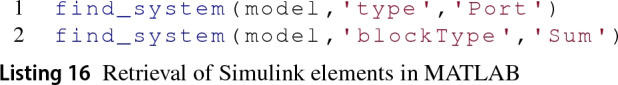


Stateflow elements are collected using the MATLAB functions in Listing 17 which act on the model handle. All Stateflow objects can be retrieved by passing the Stateflow.Object as isa parameter but subtypes (e.g. Stateflow.State) can also be passed instead. The approach to collect these from Epsilon is shown in Listing 18. 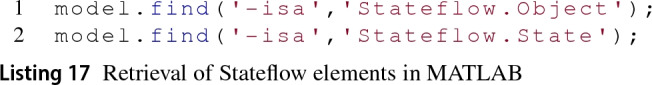

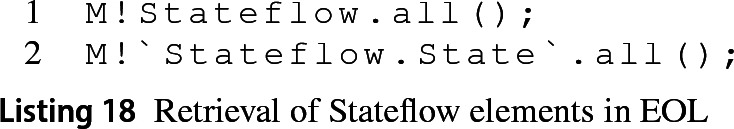


*Element Methods* The Simulink EMC adds convenience methods to its model and model elements, such as the one for linking blocks in Listing 7. Other methods are also available, such as getType(), getParent() and getChildren(). Nevertheless, MATLAB provides many more functions for its Simulink and Stateflow model elements that would be challenging to individually replicate in the EMC driver. To deal with this, when an unknown method in EOL is called on the model or its elements the following strategy is applied.

Many MATLAB functions for Simulink model and model elements have a common structure (Listing 19) which takes the model element as first argument. At the same time, model element operations in EOL are executed as instance methods and have the form shown in Listing 20. 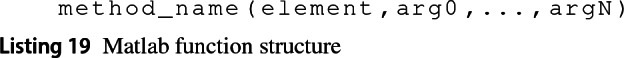

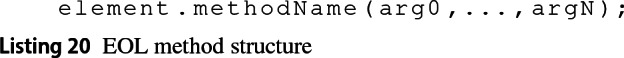


To execute non-hard-coded MATLAB functions, the Simulink driver dynamically translates the method as a MATLAB command and submits it to the MATLAB engine for evaluation. The SimulinkOperatorContributor class specifies this behaviour. As an example, consider the EOL statements in Listing 21 which would be translated to the corresponding MATLAB functions in Listing 22.
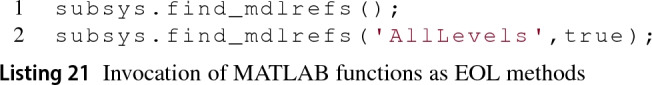

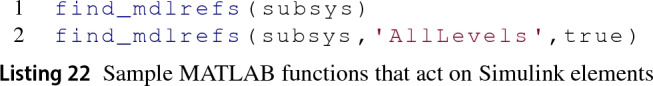


MATLAB operations acting on Stateflow elements commonly^8^ share the same syntax as EOL, except that operations with no arguments do not require brackets in MATLAB. Through the SimulinkOperatorContributor class, the Simulink EMC driver can change the translation of these functions depending on the model element kind they act upon.

#### Collection query optimisation

The Simulink driver returns lazy collections of model elements when retrieving elements by type or kind. This capability was already presented in [38]. However, performing collection and selection operations on these collections can become computationally expensive as these collections grow in size because they are performed sequentially by default. Taking advantage of some of the MATLAB functions which can perform bulk operations much more efficiently, in this work we use them on collections of Simulink or Stateflow model elements when *select* or *collect* operations involve property checks on their members.

A *collect* operation works on a collection and consists in evaluating an expression on each member of the collection to return a new collection with the evaluation results. For example, the EOL statement from Listing 23 starts on a collection of all Block elements in the model and returns a new collection with all the names of these elements. 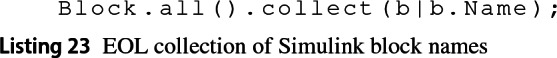


A *select* operation also works on collections and filters the collection leaving only the elements that satisfy a given condition. For example, the EOL statement from Listing 24 starts from a collection of elements of Inport type and returns a copy of the collection with only the elements named Temperature. 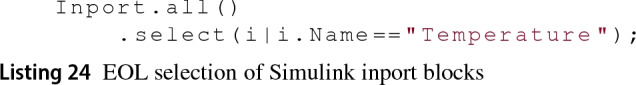


Lazy collections of Simulink or Stateflow model elements work by storing the array of model element identifiers (handles) and only constructing the appropriate wrapper (e.g. SimulinkBlock, StateflowBlock) when acting on the elements of the collection. For example, when  is invoked in Epsilon, the collection of blocks returned by the appropriate MATLAB function is an array of Simulink handles (doubles). There are operations we can compute on this array without having to resolve them into their corresponding SimulinkBlock wrapper instance. For example, we can get the number of blocks on the collection by getting the size from the array of Simulink handles. However, when select or collect operations are invoked on a lazy collection, their argument expressions are likely to involve interactions with properties from elements in the collection. As such, the lazy collections have to iterate over their elements, instantiate them in their appropriate wrapper class and evaluate their expressions.

In this paper, we have extended the implementation of the lazy collections to support the invocation of select and collect operations without having to instantiate wrapper classes for all its elements. To achieve this, the lazy collection first checks whether the operator’s expression can be optimised (i.e. has a specific form) and if so then the collection builds a MATLAB function that can use the array of Simulink handles. Currently, we optimise only those operations whose expressions can be translated into a valid bulk MATLAB statement.

For *collect* operators, we currently support simple property navigation expressions such as Listing 23. The MATLAB functions in Listing 25 are used to collect properties from collections of Simulink (line 1) or Stateflow (line 2) model elements. These functions take as first argument the array of element handles and replace the ’?’ placeholder with the property name to be retrieved.
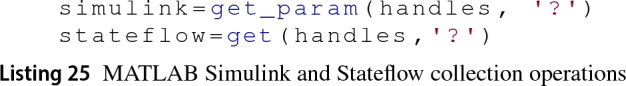


The Epsilon operator *sortBy* reuses this implementation to sort the elements on the collection after they have been collected in bulk.

The select operator optimisation for collections of Simulink model elements uses the MATLAB function in Listing 26 to perform the bulk queries. This function replaces the question mark placeholders with property-value pairs that all elements in the collection must match. When more than one property-value pair is used the function performs the logical AND operation. As such, optimised select operations in Epsilon only support expressions which involve logical AND expressions that, as in the collect case, involve simple property checks. Select operations that do not match this criteria fall back to the default sequential evaluation. 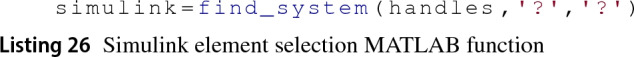


An example of a supported EOL query on a collection of Simulink model elements is shown in Listing 27. The corresponding MATLAB function submitted to the engine is shown in Listing 28, where all refers to a collection of Simulink element handles. 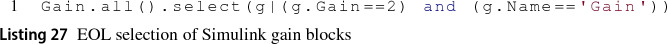

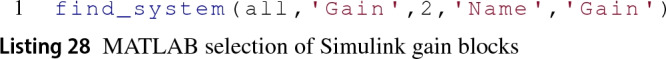


The select operator for collections of Stateflow elements delegates to the MATLAB function in Listing 29. The question mark placeholders in this function can be replaced with property-value pairs to be matched from the elements in the collection. This MATLAB function supports more fine-grained queries than the find_system MATLAB function. For example, it supports multiple logical operators (i.e. AND, OR, XOR and NOT) to join property-value pairs and also supports regular expressions for property values. 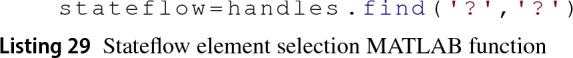


Listing 30 is an example of an EOL select operation that can be performed on collections of Stateflow elements. Listing 31 shows the MATLAB function that is constructed and submitted to the MATLAB engine, where all represents a collection of Stateflow handles. 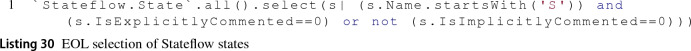

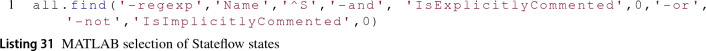


The *select* operator is reused by other Epsilon operators such as: *selectOne*, *find*, *findOne*, *reject*, *rejectOne*, *exists* and *forAll*.

### Simulink Dictionary EMC

This driver manages Simulink Data Dictionary as models. A simplified view of its architecture is presented in Group 4 of Fig. 5.

In Sect. 3.1.1, we discussed the process by which EOL statements are translated into and back-from MATLAB functions using facilities provided by the Epsilon Model Connectivity layer. Since the process to manage dictionary models is very similar, in this section we focus on the MATLAB functions involved in the translation process rather than the process itself.

*Model* Before executing an Epsilon program, the Dictionary EMC invokes the appropriate MATLAB function from Listing 32 to either create (line1) a new dictionary model file (*.sldd) or open one (line 2). After the execution of an Epsilon program and depending on the runtime model preferences, changes in the dictionary may be saved or discarded invoking the MATLAB functions from lines 3 or 4 in Listing 32, respectively. Only if specified at runtime, the dictionary may be closed after an execution invoking the MATLAB close function (line 5). 
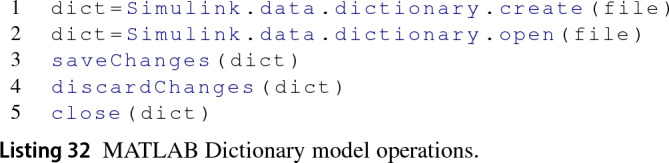


*Model Elements* As discussed in Sect. 2, the root instance of a data dictionary is the dictionary itself. In the Dictionary EMC, this instance is represented and managed by the SimulinkDictionaryModel class. The root element contains four specific sections by default: Design Data, Configurations, EmbeddedCoder and Other Data. Sections are represented by the SimulinkSection class in the Dictionary driver. Each section contains a set of entry elements which represent a key-value pair but contain more information such as their last modification date and the author of the changes. In the Dictionary EMC, the SimulinkEntry class wraps these entries.

*Retrieve Elements* MATLAB provides several functions to retrieve either sections or entries from a dictionary. The first line in Listing 33 shows how to retrieve a section from the dictionary by specifying the name of the section, in this case “Design Data”. The second line illustrates how the entries of the section can be collected. 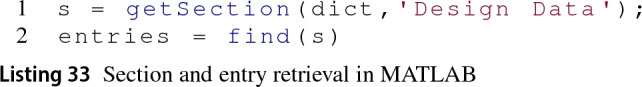


When an Epsilon program asks for all elements of type Dictionary (line 1 from Listing 34) the Dictionary EMC returns the model^9^ for convenience. For collecting the sections of a dictionary model (line 2), the Dictionary EMC invokes the getSection MATLAB function for the four sections of the dictionary and wraps each section identifier in an instance of the SimulinkSection class. As there is no method to retrieve all entries from a dictionary (line 3) in MATLAB, the Dictionary EMC invokes the find MATLAB function on each of the sections of the dictionary. Each of the entry identifiers returned is wrapped in the SimulinkEntry class of the driver. However, when the Epsilon program calls for entries of a specific section—such as in lines 4–6, the find function is only invoked once for the specific section. At the moment, this driver is unable to handle entries from the EmbedderCoder section. 
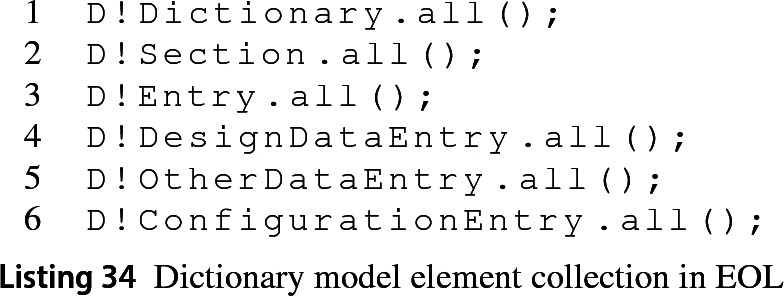


*Create Elements* There are four sections in a dictionary and we are not aware of a way to create new ones through the MATLAB API. For an entry to be instantiated, it must specify the section that will contain it, the name of the entry and its value as required by the addEntry MATLAB function from Listing 35. 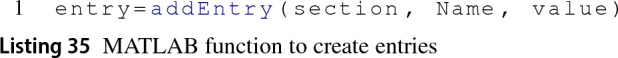


The Dictionary EMC allows the instantiation delay for entries with a specific section but no name or value, as shown in line 1 from Listing 36. This delayed instantiation saving its name and value in memory when provided and only submits the MATLAB instantiation command to the MATLAB engine when both are assigned. A delayed-instantiation approach is also applied to entries with no section. 
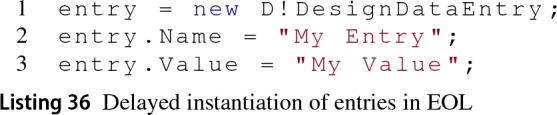


*Delete Elements* Deletion is only applicable to dictionary entries as there is no MATLAB function to delete dictionaries or sections. Listing 37 shows the corresponding MATLAB function to achieve this. 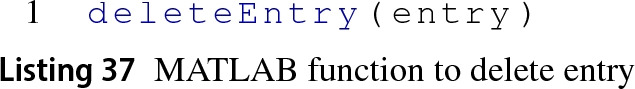


*Read and Update Element Properties* To read and write model element properties in MATLAB, dictionary, section and entry elements use the dot notation. In Listing 38, line 1 shows the retrieval of a property from the dictionary and line 2 shows how to set the name of an entry. 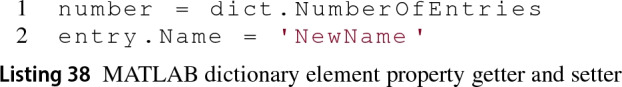


There is an exception to the dot notation and it occurs when trying to set the value property of entry objects. Getting and setting this property requires the use of special getter and setter methods as in Listing 39. 
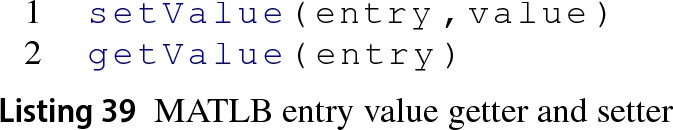


*Methods* All model elements, i.e. dictionary, entry and section, share the same syntax to invoke MATLAB functions on themselves. This notation (Listing 40) is the function name followed by the element and an arbitrary number of subsequent parameters. The Dictionary EMC is responsible for translating methods in Epsilon programs to the appropriate syntax when these act on dictionary-related model elements. 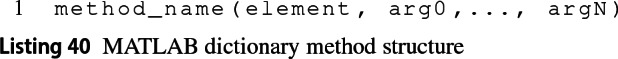


### Simulink Requirements EMC

This driver manages Simulink requirements as models. A simplified view of this driver’s architecture is presented in Group 5 of Fig. 5. As in Sect. 3.2, this section focuses on the MATLAB functions and model elements that are involved when executing CRUD operations on model elements managed by the Requirements EMC.

*Model* When an Epsilon program is about to execute, the Requirements EMC invokes the appropriate MATLAB function from Listing 41 to either create (line 1) a new requirement definition file (*.slreqx) or open an existing one (line 2). Both these functions return a handle or identifier of the Requirement Set (rs), the root model element. When an Epsilon program has finished its execution, the Requirement EMC may save the requirement set (line 3) or close it (line 4) if specified in the configuration. This corresponds to Epsilon’s model disposal process. 
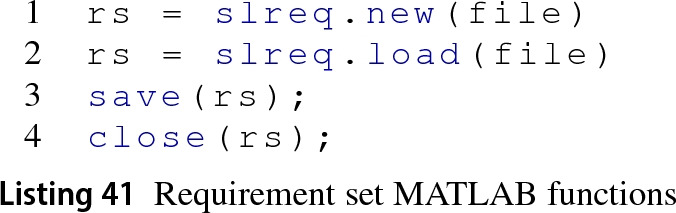


Tightly related with the requirement management process is the ability to process traceability links. MATLAB persists link information in as many link files (*.slmx) as there are models involved in the links. For example, each Simulink model (mySimulink.slx) with link traces will have a co-located link file (mySimulink.slmx) with the same name as the Simulink model. Each of these files contains all the links in which the non-link model artefact is the destination. The same applies to dictionaries and requirements. While MATLAB provides methods to load link files, when their corresponding model is loaded or opened, the set of links contained in the file is loaded in memory as well. Closing and saving one of these models has the same effect on its link set.

*Model Elements* The root instance of a requirement definition file is the RequirementSet. In the Requirements EMC, a requirement set is represented by the RequirementModel class. A requirement set contains requirement, justification and reference elements. These are handled in the driver with the SimulinkRequirement, SimulinkJustification and the SimulinkReference classes.

In MATLAB, the root instance of any link file is a LinkSet which contains all the links associated to a model artefact (e.g. a Simulink model, a requirement set or a data dictionary). As opening or loading model artefacts automatically load their link files, the Requirement driver associates all available link sets in the form of SimulinkLinkSet objects to a RequirementModel. Similarly, all the links contained by the loaded link files are managed by a RequirementModel in the form of SimulinkLink entities. However, the models of all Simulink-based drivers have a getLinks() method which returns the links associated to themselves.

*Retrieve Elements* To collect model elements from requirement definition files, MATLAB provides the functions in Listing 42. Lines 1–3 contain functions which collect elements by type (i.e. requirement, reference and justification) from the requirement set. To get elements by their subtype, the find MATLAB function receives the ReqType argument shown in lines 4–5 which can be used to retrieve requirement and reference subtypes. Justifications do not have a subtype.
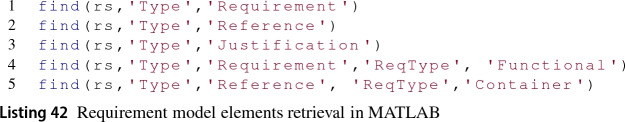


In an Epsilon program where a model R is managed by the Requirements EMC, it is possible to collect the requirements with the statement in line 1 from Listing 43. This invokes line 1 from the MATLAB functions in Listing 42 and the driver builds a SimulinkRequirement instance from the returned identifier. A similar procedure is followed for justifications and references when the Epsilon program executes lines 2–3. For collecting references and requirements of a specific subtype, the Requirement EMC needs to know if the subtype is for a requirement or a reference to pass the right value for the Type parameter in the MATLAB functions above (lines 4–5). This is why the driver requires prepending the prefix RQ_ or RF_ to the subtype (lines 4–5) to detect whether it corresponds to a requirement or a reference, respectively. 
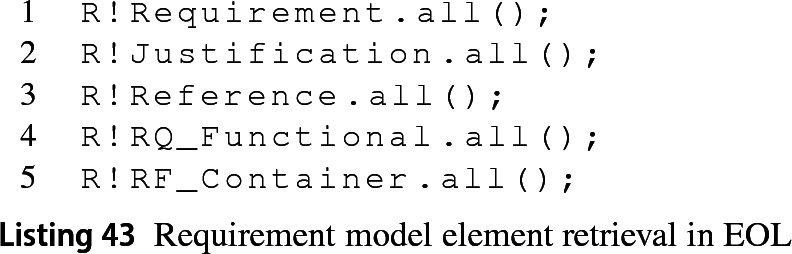


Traceability links are managed differently in MATLAB and require the use of the slreq.find MATLAB function. The first two lines in Listing 44 show how to collect all loaded link sets or links from the artefacts loaded in the workspace. Furthermore, it is possible to retrieve links of a specific type by specifying the subtype as an extra parameter (line 3). 
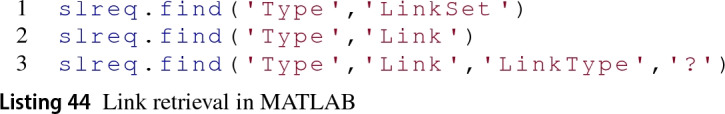
 Using the Requirements EMC, it is possible to retrieve the link sets and link elements through the use of the first two EOL statements in Listing 45. Similarly, the subtypes of links can be retrieved directly by prepending the L_ prefix to the subtype of the link. 
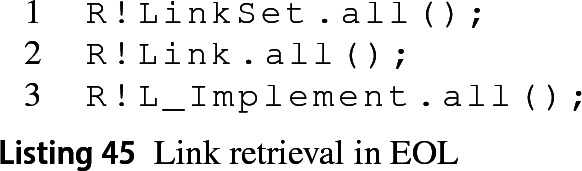


*Create Elements* MATLAB provides different approaches to instantiation for the different model elements used in requirement management. Line 1 in Listing 46 shows how to instantiate a requirement in the loaded requirement set (rs) by invoking the add MATLAB function. To create justifications as illustrated in line 2, MATLAB provides the addJustification function which takes the requirement set as input. Similarly, to create a reference the add function requires the parameters Artefact and Domain to distinguish a reference from a requirement. 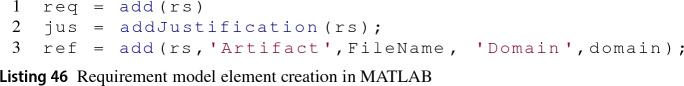


Following the same logic as other Simulink-based drivers described before, to create instances of type requirement, justification and reference in Epsilon programs it suffices to invoke statements such as those in lines 1–3 in Listing 47. The Requirements EMC driver allows the direct instantiation of requirement subtypes (as in line 4) because the add MATLAB function can also receive additional key-value pairs to configure the requirement at instantiation. Instantiation with subtype is currently not supported for references in the Requirements driver. 
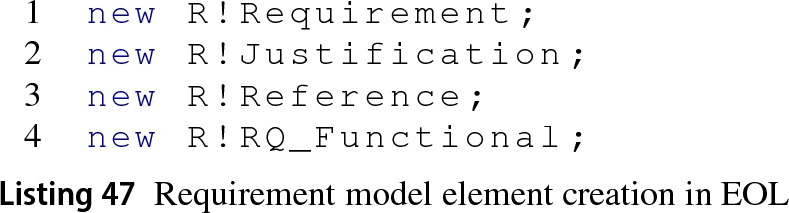


In MATLAB, links require source and destination artefacts to be instantiated. The Requirements EMC allows the instantiation of the SimulinkLink class with no source and destination artefacts as long as these are provided later. At that point, the class prepares the MATLAB function in Listing 48 with the appropriate values and submits it for evaluation to MATLAB’s engine. Direct instantiation of link subtypes is not currently supported by the driver, but the subtype can be assigned as a property. 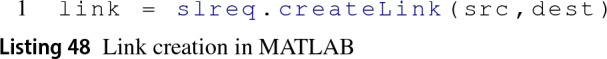


*Delete Elements* The remove MATLAB function is used to delete link, reference, justification and requirement elements. The Requirements EMC submits the MATLAB function in Listing 49 when a wrapper instance for a link, justification, reference or requirement is to be deleted in an Epsilon program. 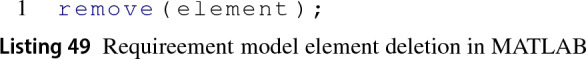


*Read and Update Element properties* Reading and writing properties of requirement, reference and justification model elements can be achieved by invoking the MATLAB functions in Listing 50 with the appropriate property key and values. In contrast, to update or modify properties of the requirement set (i.e. the model), link sets and links, MATLAB offers a more direct approach where the values are read or modified using a dot notation as in Listing 51. 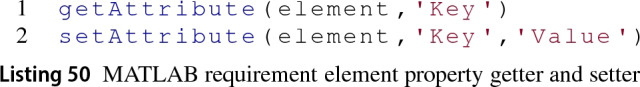

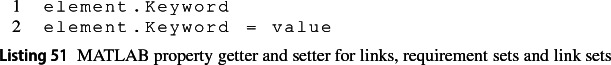


*Methods* The syntax of functions acting on requirement and link model elements follow the pattern in Listing 52. As done in other Simulink-based drivers, the Requirements EMC provides an operation contributor which translates method calls on model element instances in EOL to the appropriate MATLAB syntax. For example, the EOL methods in Listing .53 are submitted for execution to the MATLAB engine as in Listing 54. 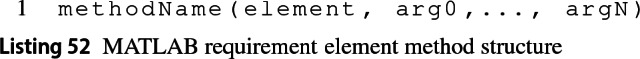

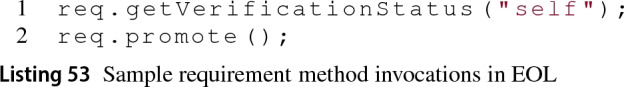

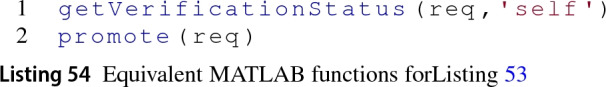


## Multi-model example

**Fig. 6:**
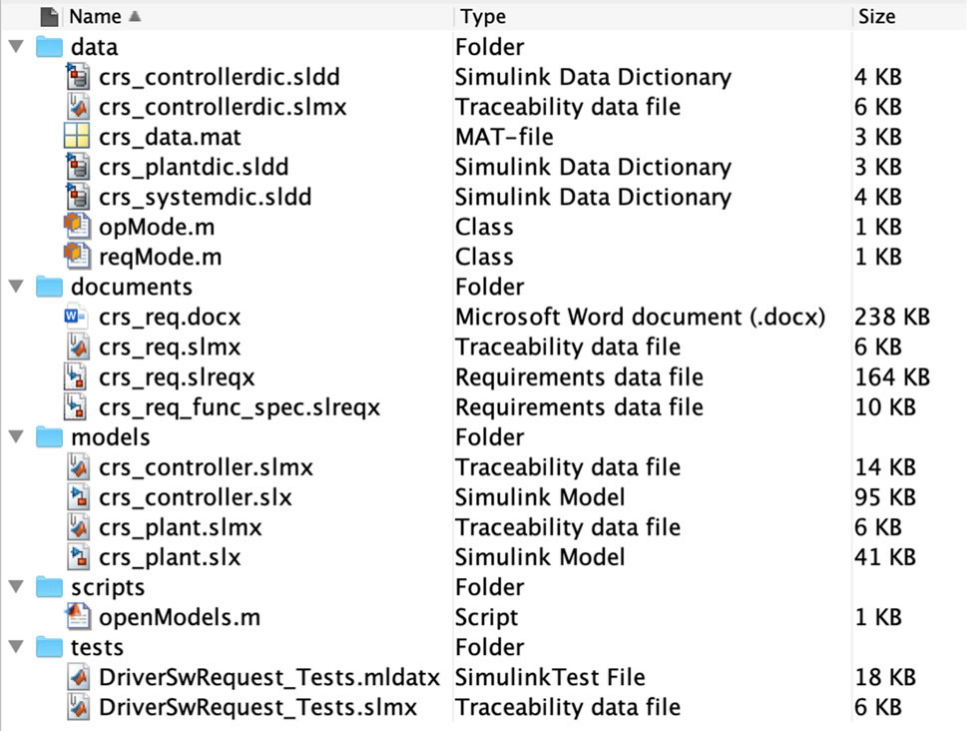
MATLAB Project Structure

**Fig. 7:**
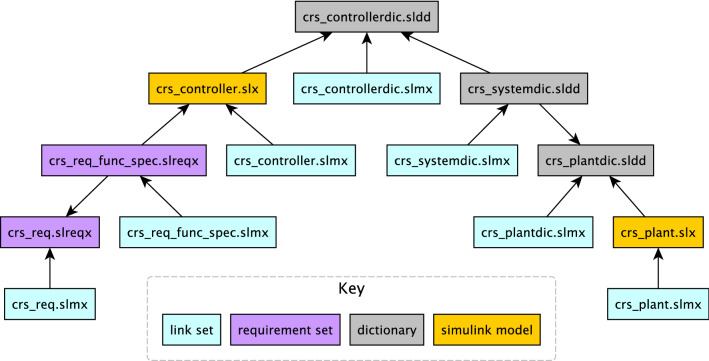
Model dependencies

**Fig. 8:**
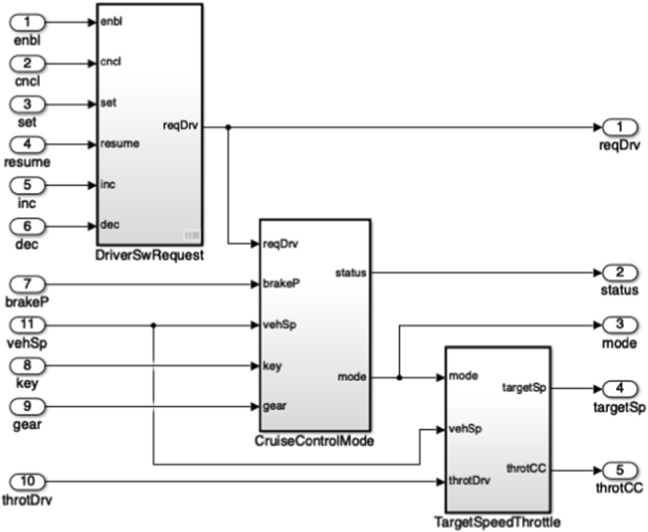
Simulink model: crs_controller.slx

**Fig. 9:**
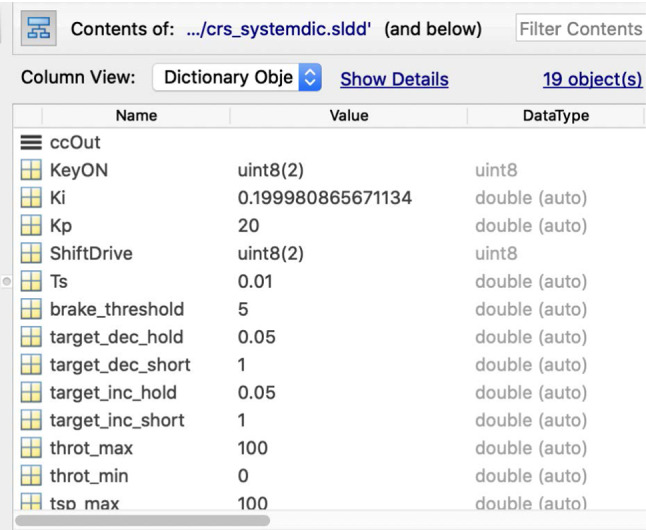
Dictionary: crs_controllerdic.sldd

**Fig. 10:**
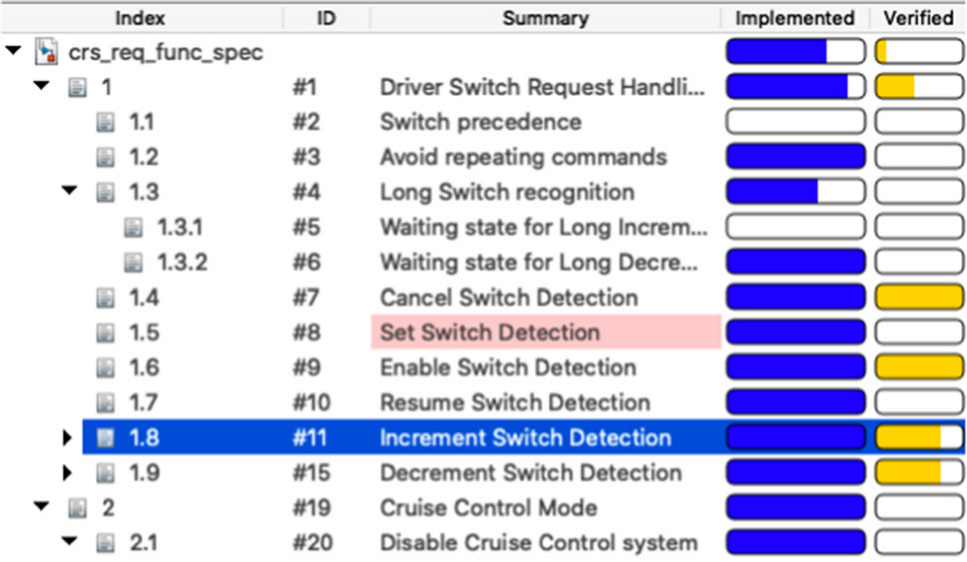
Requirement set: crs_req_func_spec.slreqx


To showcase how we extract knowledge from Simulink related models with the drivers presented in the previous section, we now introduce a running example that works on a sample MATLAB project [27] which represents a cruise control system. This project is composed of several files out of which there are two Simulink models, two requirement set files and three data dictionaries. Each of these files has a corresponding traceability link file as illustrated in Fig. 6. Their interdependencies are illustrated in Fig. 7. In this example we will only be working with the crs_controller.slx Simulink model (Fig. 8), the crs_controllerdic.sldd dictionary (Fig. 9) and with the crs_req_func_spec.slreqx requirement set (Fig. 10).

Our aim with this running example is to analyse this project, in particular regarding traceability well-formedness and unused/unlinked elements. As such, we now present three analysis scenarios which involve different combinations of Simulink, dictionary and requirement models. The first scenario consists in identifying Simulink blocks with no traceability links. The second scenario consists in counting how many requirements are barren and orphan. The third scenario checks whether Simulink block configuration variables are present in a dictionary as entries.

*Simulink elements with no traceability* Listing 55 shows an EOL program that can be used to identify the Simulink blocks which have not been associated with traceability links. When this program is evaluated against a Simulink model, the EOL execution engine retrieves all Simulink blocks in the model. The engine then iterates over these elements to evaluate the MATLAB function slreq.outLinks which returns a list with their outgoing links. The select operator ensures that only those elements with no links for a block are stored in the unlinked variable. Because in this case no block on the model has incoming links, the program only needs to check for outgoing links to determine that a block has no traceability information. Lines 2 and 3 proceed to compute the path location of the blocks with no links and the number of elements found with no traceability, respectively.

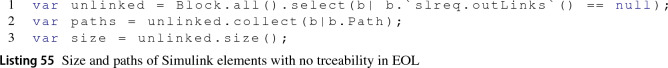


In this program, the execution of the select operator is not optimised (see Sect. 3.1.2) as it involves a method invocation and not a property check. In contrast, the execution of the collector is indeed optimised. The EOL method ‘slreq.outLinks‘() is resolved at runtime and submitted to MATLAB as slreq.outLinks(handle).

We observe that 390 blocks out of 454 have no traceability information in the Simulink model crs_controller.slx.

*Barren and orphan requirements* Requirement analysis commonly involves the identification of barren and orphan requirements. The definition of barren or orphan varies across projects. In this example, we define a barren requirement as a top requirement with no outgoing links while an orphan as a leaf requirement with no incoming links. One way of identifying a top requirement is to check if it has no parent requirement while to identify a leaf requirement we check if it has no children. In this example, we use a different approach in which a top requirement is one of type *container* while a leaf requirement is of type *functional*.

Listing 56 shows an EOL program for computing barren and orphan requirements. This EOL program is executed on a Simulink requirement model. Line 1 computes the barren requirements by collecting the requirements of container type and then filtering those which have no outgoing links. Line 2 computes the orphan requirements by collecting all the functional requirements and then filtering those with no incoming links. The direct lookup of requirements of type container or functional is a facility provided by the Simulink requirements driver.
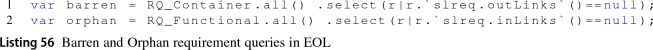


In this particular case, none of the select operators are optimisable because they do not check any property value.

The execution of this analysis on the requirement set provided by crs_req_func_spec.slreqx indicates that all requirements of type container were barren. In contrast, only 16 out of the 66 functional requirements were orphans.

*No missing entry definitions* When using dictionary entries to configure blocks on a Simulink model, a common analysis involves identifying blocks that specify configuration variables which point to non-existing dictionary entries.

In the Simulink model crs_controller, there are blocks which are configured with entry values from the dictionary crs_controllerdict. This is the case of saturation blocks which have their upper and lower limit values set-up from this dictionary. Similarly, 5 out of 13 blocks of type constant specify their value from an entry in the dictionary.

Listing .57 shows an EOL program for identifying blocks with configuration values pointing to non-existing entries. In this program, only the select operation of line 2 is optimised as it performs a property check on a collection of Simulink model elements. This program must be executed against the dictionary model (D) and the Simulink model (S). The first line of this program collects all the entries from the dictionary model. To check whether the constant blocks are referencing entries that do exist, line 2 starts by retrieving all blocks of type constant and then filters those which will inherit its value from a dictionary in the select operator. The last part of this query rejects all those elements which have no match in the list of entries from line 1. Saturation blocks follow a similar approach to identify the blocks which are using non-existent configuration values. Line 3 presents the query for these blocks which starts by retrieving all elements of type Saturate. Assuming that by design we expect blocks of this type to use values from a dictionary, the next step consists in checking whether the upper and lower limit values are referencing entry names present in the dictionary. 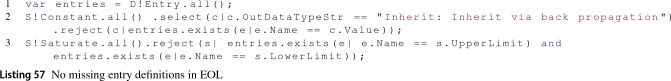


The execution of this program on the Simulink model and data dictionary from the running example confirms that there are no blocks using configuration values with no matching dictionary entries

With the use cases above, we have demonstrated how the drivers can be used to perform analysis with information from the Simulink-based models. We have highlighted the situations in which collection optimisations can be invoked and demonstrated that multiple models can be run simultaneously.

## Evaluation

This section presents a two-part evaluation of the Simulink–Epsilon drivers. The first part (Sect. 5.1) consists of an experiment that compares the performance of managing Simulink models directly via MATLAB functions or building an intermediate EMF representation with an upfront model-to-model transformation. This experiment was first published in previous work [38]. The second part of the evaluation is presented in Sect. 5.2 and compares the performance of collection operators executed on collection of Simulink and Stateflow model elements using the query optimisations described in Sect. 3.1.2.

### Experiment on Simulink models

This section evaluates the execution-time performance of two approaches to bridge MATLAB/Simulink models in a model management framework. The first approach consists in using the Simulink model driver to manage models in the Epsilon model management framework. The second approach uses Massif facilities to transform Simulink models into an EMF-compatible representation. Since Epsilon provides an EMF driver able to read and write arbitrary EMF-based models, we use it to manage those produced by Massif in the second approach. In the following, we refer to the first approach as *live*—since it directly manipulates the actual Simulink model, and to the second one as *Massif/EMF*—as it uses the Massif’s import facilities to produce their EMF-compatible representation.

Epsilon supports model element caching through an abstraction that both the Simulink model driver and the EMF driver reuse. We evaluate both approaches with these facilities enabled and disabled. Note that at the time of this experiment, the query optimisations on Simulink and Stateflow elements had not been implemented.

#### Experiment set-up

In order to evaluate the model management of Simulink models through both approaches, we compare the performance of their model validation process applied on large Simulink models. We have selected a model validation process as a representative model management operation although other operations such as model-to-model or model-to-text transformations could have been used instead.

*Validation process* This process is based on the execution of EVL invariants that validate structural properties of the models. EVL has a dedicated engine that consumes an EVL validation script and any number of models provided by Epsilon drivers of arbitrary modelling technology at runtime. An example of an EVL script is shown in Listing 58. This script starts by specifying the context in which the invariants are to be executed, in this case all elements of kind Block. Invariants may be of type *constraint* or *critique* depending on the severity level of a failed compliance. Line 2 of the script shows the declaration of an invariant of type *critique* with name BlockNameIsLowerCase. Invariants declare their validation *check* as an EOL statement, which in this case (line 3) verifies that the name of the element is lowercase. The self reserved word is a reference to the current model element the invariant is acting on. If a given block fails the check statement, then *fix* elements become available if present in the invariant declaration. In the script, the fix in line 4 updates the element name to lowercase as specified in the *do* environment (line 7). The fix title (line 5) is just informative. 
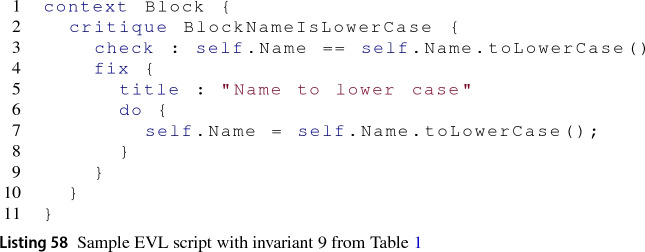

**Fig. 11:**
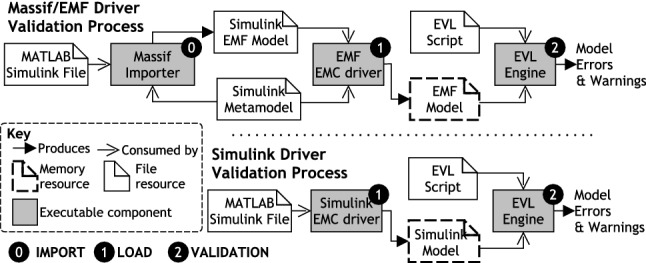
Execution process for model management programs, in this case, a model validation with EVL

Before the EVL engine can execute the model validations, the models must be loaded. When the EMF driver is used to process an EMF model, the model loading stage consists in the registration of meta-model packages and creating an in-memory representation of the model. When the Simulink model driver is used to process a Simulink model file, the model loading stage consists in establishing the connection with the MATLAB engine and requesting the model to be loaded there.

In the following, we consider the model loading and validation execution as two different stages of the validation process. The overall validation process for each approach is captured in Fig. 11 where loading and validation are represented by stages 1 and 2, respectively. In the Massif/EMF approach, we consider the transformation of the model (from Simulink to EMF) as an additional stage of the validation process (Stage 0 in Fig. 11). We refer to it as the import stage after the Massif facilities that enable this transformation.

The implementation of the Epsilon drivers and the structure of the meta-model used in the EMF driver affect the way the model is navigated in EOL-based programs. Consequently, the EVL validation script cannot be reused *as-is* across approaches. To illustrate this, consider an EOL program that retrieves the PortDimension property of a block model element. When executed on a model managed with the Simulink model driver, the EOL statement from Listing 59 is able to retrieve this property from an element of type block. 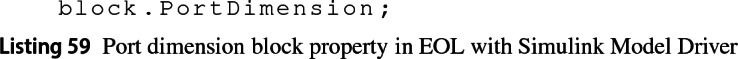 In contrast, when using the EMF driver with the Massif meta-model, the statement needs to be adapted (as in Listing 60) because the Block class in the meta-model does not have a PortDimension attribute but instead has a parameters attribute containing a set of Property elements, one of them with the PortDimension identifier. 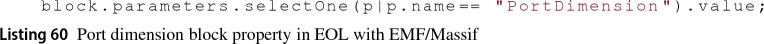


In this experiment, we measure the execution-time performance of the different stages of the validation process, i.e. (0) Simulink-to-EMF transformation, (1) model loading and (2) model validation. Notice that: Stage 0 is only applicable to the *Massif/EMF* approach; Stage 1 is applicable to both approaches; and Stage 2 is applicable to each approach with both the Epsilon caching facilities enabled and disabled.

Each stage of the validation process was executed 20 times with 5 warm-up iterations for each model. We used the Java Microbenchmark Harness (JMH) [33] tool to run these experiments on a quad core Intel Core i5-7200U CPU @ 2.5 GHz with 16GB of RAM. The Java Virtual Machine (64-Bit) was provided with up to 10GB of memory and ran Java 8 on JDK 1.8.0_152. All EMF-compatible models were generated using the *shallow* mode of the Massif import facilities which does not process external model references. The validation scripts and the Simulink models that were used in our experiments can be found in the examples of the Epsilon project [10].

**Table 1:** Evaluated invariants

#	Kind	Context	Description
1	PropertyCheck	Goto	TagVisibility property is local
2	NavigationAndFilter	From	There is a Goto block in scope with the name of the GotoTag property
3	PropertyCheck	Inport/InPortBlock	PortDimensions property should not be inherited ($-1$)
4	PropertyCheck	Outport/OutPortBlock	Description property is not null or empty
5	NavigationAndFilter	SubSystem	ForegroundColor property is green for all connected Inport blocks
6	TransitiveClosure	SubSystem	Subsystem is no more than three levels deep
7	VertexConnectivity	SubSystem	All outports are connected
8	LoopAbsence	SubSystem	No feedback. Outports do not connect to the same subsystem
9	PropertyCheck	Block	Block’s name is in lower case

*Validation scripts* Equivalent EVL scripts are used to evaluate each approach. Each script consists of 9 invariants (see Table 1) intended to exercise the model (e.g. using different operations or navigation strategies) through typical query language features [41] performed on signature model element types [2]. The scripts are equivalent to the best of our knowledge as they are using (a) equivalent EVL *contexts* which may vary in naming across approaches (e.g. Inport vs. InPortBlock), (b) equivalent model element navigations (such as the PortDimension property discussed above), and (c) equivalent way in which the constraint checks and guards are prescribed. In Table 1 the *Kind* column refers to type of query check inspired on well-formedness constraint categories used by the Train Benchmark [41], and the *Context* column refers to the EVL context, that is, the model element types on which the invariant is executed. Stateflow blocks were not included in the validation scripts as Massif does not support them.

The validation scripts for the *live* approaches used 96 lines of code (LOC) and that for the *Massif/EMF* approach used 110 LOC. The body of the invariants was written in the same number of lines for both approaches (89 LOC) and the extra lines were related to helper operations.

*Model selection* We used BigQuery [13] to find in GitHub publicly available Simulink files (*.slx) larger than 1 MB.^10^ Out of the 70 models found, we selected the first 7 models that could be translated into EMF in under 2 h using Massif’s import facilities. Table 2 shows the number of model elements of each type used in the validation. The number of block elements on the models ranged from 8628 to 9536. Due to their inaccessibility, we did not process any libraries in any approach.

**Table 2:** Number of elements per type by model size

Size	Block	Inport	Outport	Goto	From	SubSys.
1.112	8785	1373	1177	69	103	717
1.131	8628	1372	1167	62	93	740
1.133	8645	1372	1167	62	93	740
1.134	9536	1489	1269	38	57	861
1.135	8645	1372	1167	62	93	740
1.138	8651	1376	1177	62	93	745
1.141	8634	1374	1156	67	99	714

#### Results

**Fig. 12:**
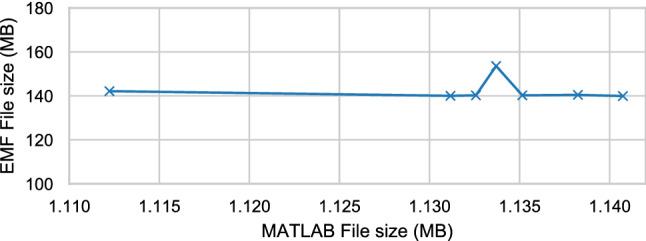
Size of the imported EMF models against the original MATLAB files

All invariants were executed in the same number of model elements for all approaches. Similarly, the results of the validation reported the same number of failed constraints on all approaches. The file size of the EMF models produced by the import stage are displayed in Fig. 12, plotted against the size of the original MATLAB file.

**Fig. 13:**
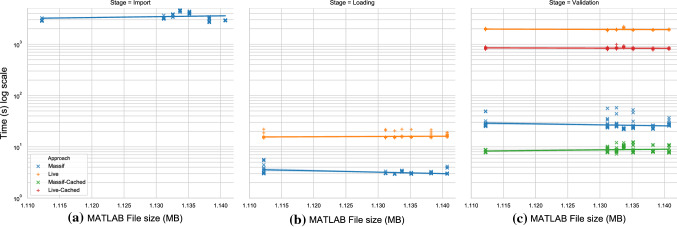
Execution-time duration in log-scale against MATLAB/Simulink model file size per stage of the validation process

Figure 13 shows the execution time of each stage of the model validation process (in seconds and logarithmic scale) against the size of the MATLAB Simulink model files (in MB). Sub-figure (a) displays the distribution of Massif’s *import* task (Stage 0) which transforms Simulink models into an EMF-compatible model. Similarly, Sub-figure (b) displays the time distribution of the model loading task (Stage 1), required by both the EMF and Simulink model drivers. Sub-figure (c) displays the time distribution of the model validation task (Stage 2) for both approaches with and without caching.

Figure 13 shows that most of the performance overhead of the Massif/EMF approach happens at the import stage while most of the Simulink model driver overhead happens at the validation stage. The import stage of the Massif/EMF approach took between 4486 and 2911s to finish. The Massif/EMF approach achieved the loading stage in 2.95–3.63 s, while the Simulink model driver achieved it in 15.5–16.5 s. The live approach was approximately 1 order of magnitude slower at the loading stage. In the validation stage, the Massif/EMF approach took between 22.4–28.9 s, while it took the Simulink model driver 1877–2098 s to complete. With caching facilities enabled in both drivers, the Massif/EMF approach took 8.10–10.2 s, while the Simulink model driver took 816–882 s to finish. With and without caching, the live approach was approximately 2 orders of magnitude slower at the validation stage. The caching facilities improved the performance in the validation stage by 54.4–72.0% in the Massif/EMF approach and 55.3–58.0% in the live approach.

**Fig. 14:**
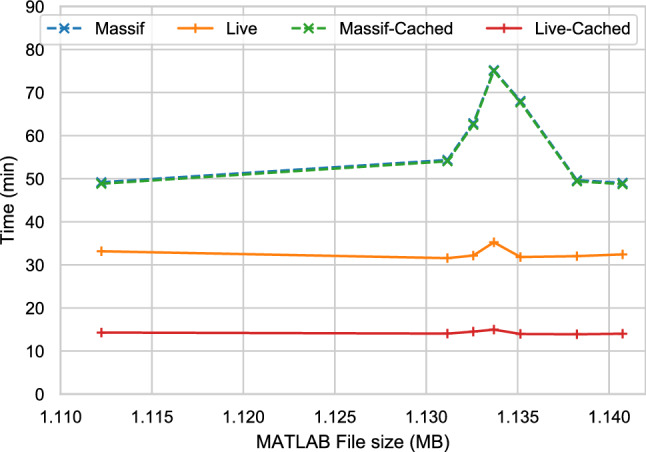
Total execution-time duration (log-scale) against MATLAB file size. *Note that Massif and Massif-Cached overlap.*

Figure 14 shows the whole validation process execution-time (in minutes) calculated using the sum of averages of each stage for each approach with and without caching. By comparing this overall process, we observe that the live approach improves the performance of the Massif/EMF approach by taking 70.7–80.0% less time when caching is enabled and by 32.6–53.2% with no caching.

In Fig. 12, we observe that the size of the EMF model produced by Massif is much larger than the original MATLAB/Simulink (.slx) files. This is partly due to *.slx being a compressed file format. As Table 2 shows, the size of the MATLAB/Simulink file is not directly proportional to the number of Block^11^ elements in the model. In contrast, the size of the EMF model file seems to be related to the number of block elements, which would explain the peak on the EMF file size with the MATLAB/Simulink model with the largest number of block elements.

#### Discussion

In this experiment, we focused on a program that only reads large Simulink models. We intended to investigate the performance of using of both approaches with large models. In this particular subset of models, our experiment shows that the overhead of the Massif/EMF approach lies on the upfront model transformation, whereas for the Simulink EMC, it lies in the complexity of the model management program. In contrast, the actual execution of the program with the EMF driver works much faster than with the Simulink EMC driver. This is partly due to the full model being loaded in memory and potential internal optimisations of the mature EMF driver.

Intense querying is a scenario for which the EMF approach is more suitable, as the communication with MATLAB is expensive, and our experiment shows the clear advantage that the EMF driver has over our Simulink implementation. However, our experiment also shows the non-negligible impact that the importing stage has over the overall execution. Choosing one approach over the other is a matter of determining the size of the model, understanding the purpose of the model managing program and being aware of constraints such as performance or model coverage. For example, it is likely that large models will incur in expensive import procedures with Massif. Whether this is a sensible cost depends on the number of times the import is to be executed, the available time, the model management framework to be used, i.e. if it only supports EMF and the range of operations to be performed (e.g. do they require Stateflow blocks?). To avoid the cost of the import process on continuously evolving models, a practitioner may choose to manually replicate modifications in the Simulink model in the already imported EMF copy; however, this would be an error-prone activity.

With the same large models, our implementation avoids the import/export procedures when the models are evolving, e.g. changing property values, adding new blocks or removing blocks. Indeed, intense querying is not the best use scenario for our driver as demonstrated by the experiment. With the knowledge of the new query optimisations, the validation scripts used in the experiment could be rewritten to take advantage of these optimisations in order to reduce cost of the validation stage.

In Section 5.2, we show how the driver can be used to generate Simulink models. Further experimentation would be needed to evaluate how the approaches cope with programs that not only read the models but also modify them. Validation scripts in EVL can also feature fix constructs that invoke EOL expressions on the elements that do not pass the constraints. While we have not evaluated this, we can anticipate that the validation step with fixes would require little additional time for both the Simulink EMC driver and the EMF driver. The difference would be that the overall validation process with Massif/EMF would require an additional step to generate the modified Simulink model from the modified EMF which could potentially be just as expensive as its import procedure.

#### Threats to validity

We selected a validation program as a representative model management operation to compare both approaches. As indicated in the *Validation scripts* paragraph, the invariants used in the experiments were intended to exercise the models in similar ways in both approaches by means, for example, of interacting with the same types of elements and navigating properties in similar ways. As such, the invariants were not intended to be representative of validations performed in industry, although some were inspired by industrial cosmetic checks. Validations performed in EVL can be seen as complementary validations as Simulink models can go through custom validation checks within MATLAB using its Model Advisor tool.

Our evaluation only tested the performance of a single model management language (EVL). Performance results may vary across other types of model management programs and also for different EVL programs. Moreover, the validation script was limited to read-only operations.

The sample of models may not be significant but was limited by the 2-hour cap imposed to the import stage. Our experiments would benefit from more diverse models with a broader range of sizes and more varied constraints.

There may be hidden differences in the implementation of each driver (EMF vs Simulink) such as internal optimisations which do not make them entirely comparable. However, for the purpose of this experiment, both driver implementations were considered black boxes.

Large and complex models can be built by referencing multiple models persisted in small files. Our decision to use large models allowed us to skip the model reference processing by ensuring that a single model contained the most model elements. Additional metadata other than model elements, such as images, can contribute to the model file size without affecting its complexity. We have not measured the impact of the meta-information in the file size, but this is mitigated by indicating the number of model elements that were present in each file.

### Experiment on collection queries

We have designed an experiment that evaluates the performance of the collection operator optimisations presented in Sect. 3.1.2. The research question is whether these modifications improve the performance of select- and collect-based operators when executed on collections of Simulink or Stateflow elements of different sizes. All resources required to reproduce the experiment are available under the Epsilon project [9].

#### Experiment set-up

This experiment includes the evaluation of EOL queries on collections of Simulink and Stateflow model elements. We execute each query on four models with a similar structure but with different number of model elements that grow exponentially. For each query and model, we observe how the use of the query optimisations on collections affects the execution performance.

As we need to have control over the number of elements of a given type on each model, we decided to generate the test models. As such, the models share a similar structure but have some variability which is described later in the paper. While the generation script is not part of this evaluation, it serves to demonstrate the write capabilities of the Simulink model driver.

*Model Generation Process*

A boiler control system can be designed using an on/off closed-loop control. Closed-loop control systems are very common and they can be designed and simulated using the Simulink environment. Furthermore, on/off controllers are easy to model as state machines which can be designed using MATLAB’s Stateflow environment. Since boiler systems can contain both Simulink and Stateflow model elements, we use them at the core of our model generation process.

**Fig. 15:**
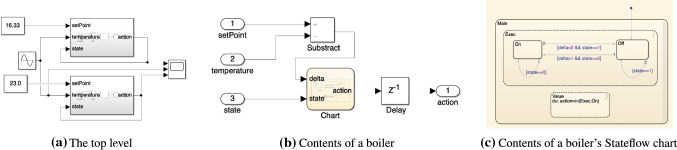
Structure of generated Simulink models

The model generation process consists in producing a number of contrived components with different set points^12^ all receiving the ambient temperature from a pulse generator and displaying their status in a scope. In order to scale our experiment, each model has a different number of boilers which grow exponentially (base 3) and the value of their set point is spread out so that each has a different value within their operational range. At the same time, each boiler has only one pulse generator and scope. Four models were generated in total.

Figure 15a illustrates the root level of the model where all boilers receive as input the ambient temperature from a pulse generator and display their operational state in a scope. The set point of each boiler is represented by a block of type constant with the temperature value. The internal structure of a boiler is illustrated in Fig. 15b. Each of them has three input ports and an output port. The inport that receives the set point is compared with the current ambient temperature using a block of type substract, whose output goes into a Stateflow chart. The contents of a chart are illustrated in Fig. 15c. The chart computes the logic to go from state ON to state OFF and produces a signal that decides whether it is required to turn on or off the boiler. The action which results from the chart logic goes into a delay which represents the time taken for the real boiler to respond to the signal. The delayed signal is displayed in the topmost scope and the one which is used as feedback on the boiler subsystem and chart.

*Queries* The list of EOL statements to be evaluated is presented in Listing 61 where line numbers are used as query identifiers. These queries were designed to demonstrate both the usefulness of retrieved information from the boiler model, and the complexity supported by the query optimisations on collections. Four of these statements are executed on collections of Simulink elements, while the other four are executed on collections of Stateflow elements. The queries use EOL select and collect operators both in plain form and derived form, e.g. exists, sortBy, reject, forAll. While most of the queries use single operators that evaluate one-argument predicates, Query 6 uses two operators (select and forAll) and Query 8 evaluates a three-argument predicate. 
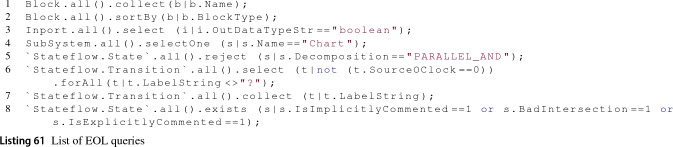


Query 1 is used to retrieve the names of all Simulink blocks in the model, including those contained in the boiler subsystems. Query 2 sorts all these blocks by their block type. Query 3 acts on blocks of Inport type, i.e. input ports 1 to 3 in each boiler subsystem (Fig. 15b), and filters those of Boolean type, i.e. port no. 3 which handles the boiler state. Query 4 acts on subsystem blocks which include the boilers and the chart blocks and selects the first element with the name “Chart”. Moving on to Stateflow elements, the list of non-parallel states is retrieved with Query 5 using the reject operator. Query 6 starts by filtering out default transitions, i.e. those with no source state, and then checks if they have all been assigned a non-default name using the exists operator. In a similar fashion to Query 1, Query 7 retrieves the labels attached to all transitions in the model. Finally, Query 8 checks for malformedness across Stateflow states by checking whether they are explicitly or implicitly commented or if they have bad intersections.

*Model population* Our experiments evaluate the 8 EOL statements on four different models. Each evaluated EOL statement starts from a collections of model elements of a given type. These model element collections may contain Simulink elements of type Block, Inport or SubSystem; or Stateflow elements of type Stateflow.State or Stateflow.Transition. The number of elements of each type in the different models is presented in Table 3.

**Table 3:** Number of elements per type on each model

	Model 1	Model 2	Model 3	Model 4
Block	47	137	407	1217
Inport	15	45	135	405
Stateflow.State	15	45	135	405
Stateflow.Transition	15	45	135	405
SubSystem	6	18	54	162

*Infrastructure* In the experiment, each EOL statement was executed 20 times with 5 warm-up iterations on each model. The Simulink model driver caching facilities were not used. The experiments were executed on an 8-Core Intel Core i9 CPU @ 2.3 GHz with 16 GB of RAM. The Java Virtual Machine (64-Bit) was provided with up to 2 GB of memory and ran Java 8 on JDK 1.8.0_231.

#### Results

In both optimised and non-optimised executions, all queries were executed on the same number of elements and yielded the same results.

**Table 4:** Mean query execution time in seconds and percentage of time spent sending commands to MATLAB and awaiting a response

		Duration (s)				MATLAB Communication (%)			
Q	Opt	Model 1	Model 2	Model 3	Model 4	Model 1	Model 2	Model 3	Model 4
1	Off	0.15	0.38	1.06	3.39	94.74	96.64	97.32	97.61
	On	0.00	0.00	0.01	0.01	76.07	75.24	76.47	73.99
2	Off	0.20	0.55	1.73	4.96	97.21	97.70	97.95	97.79
	On	0.09	0.24	0.86	2.23	96.92	98.10	98.18	98.50
3	Off	0.06	0.14	0.35	1.08	95.11	96.69	97.06	97.24
	On	0.00	0.00	0.00	0.01	75.44	73.23	68.67	59.88
4	Off	0.02	0.06	0.16	0.47	93.60	96.77	97.44	98.06
	On	0.01	0.01	0.01	0.01	86.22	84.45	81.85	75.24
5	Off	0.12	0.39	1.93	14.87	95.59	97.05	98.13	99.07
	On	0.01	0.02	0.12	0.91	89.03	95.15	98.88	99.78
6	Off	0.70	2.28	8.33	38.49	98.80	98.97	99.15	99.39
	On	0.03	0.04	0.16	1.25	95.00	97.09	99.06	99.83
7	Off	1.03	3.13	10.17	38.17	99.31	99.38	99.49	99.58
	On	0.02	0.04	0.14	1.02	95.63	97.42	99.09	99.81
8	Off	2.72	8.50	28.04	109.40	99.49	99.49	99.55	99.60
	On	0.03	0.05	0.15	0.98	96.76	97.79	99.17	99.82

Column Q indicates the query number, while column Opt indicates whether the optimisations were enabled

**Fig. 16:**
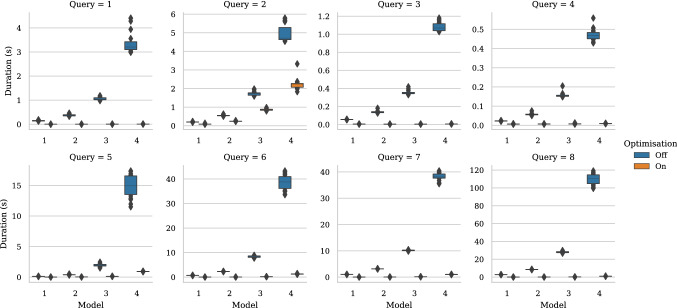
Distribution of the query performance on the models with optimisations off (left/blue) or on (right/orange)

The mean execution time of each query is presented in Table 4 under the Duration section. This section compares the time (in seconds) taken by each of the models with and without the collection operator optimisations. The iteration distribution on the four models is presented in the box plot of Fig. 16. This figure compares the distribution with optimisations enabled (right/orange) and disabled (left/blue) for each model. Note that subplots do not share the *y*-axis in order to have a closer look at the distribution per query.

Regardless of the collection size, all queries with optimisations enabled outperformed those which did not use them, between 50 and 99%. Table 5 summarises the performance improvement percentage that optimisations achieved on the different models and queries.

**Fig. 17:**
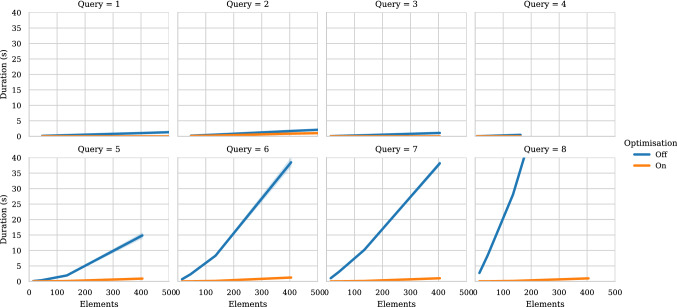
Performance of queries, with and without optimisation, against the number of elements in the models

Another view of the results is presented in Fig. 17 where the mean execution time per query is plotted against the number of model elements that the query acted on. The *y*-axis in this view has been capped at 40 seconds and only Query 8 went above this limit.

Additionally, Table 4 shows (under the MATLAB Communication section) the percentage of execution time that was spend sending or receiving information to/from MATLAB. Overall, this section shows that without operator optimisations the impact of the communications with MATLAB lies above 93%, whereas with optimisations, the impact can be reduced to 59% in some queries although remaining high (e.g. 99%) in others.

#### Discussion

The first four queries acted on Simulink elements, while the last four acted on Stateflow elements. Non-optimised queries were more expensive on Stateflow elements than on Simulink elements regardless of the complexity of the evaluated expression. In particular, consider queries 1 and 7 which are comparable as they both invoke a collect operation that gathers a single property value but work on Simulink blocks and Stateflow transitions respectively. Despite the fact that in Model 4 query 1 acts on 1217 blocks while query 7 only on 405, query 7 is much more expensive than query 1 (without the optimisations). Since more than 98% of the execution time of Stateflow queries without optimisations is spent on the MATLAB exchange, a reasonable explanation for this difference is that MATLAB has more efficient indexes for Simulink blocks.

Based on preliminary observations, executing the functions that the driver generates in the MATLAB console is much faster than through its Java API for both the optimised and non-optimised implementations. In light of the impact that reducing the number of exchanges with the MATLAB Java API has, future work will involve investigating optimisations of more complex collect- and select-based arguments so they can be transformed into a single complex MATLAB function that only requires to be sent once.

To take advantage of these optimisations, the model management programmer should be aware of the particular operations that have been optimised to write the programs accordingly. A difference with the Massif/EMF approach is that in that approach there are no particular optimisations to be aware of.

#### Threats to validity

The models used in the experiment had a similar internal structure as it enabled us to focus on the impact of the number of model elements that the queries acted upon. From this experiment, it is unclear to what extent the structure of the models affects the performance.

We chose a range of collection queries that were sufficiently varied and which could be optimised. We recognise that our evaluation could be complemented with more queries evaluating a broader range of expression forms.

## Observations and lessons learned

This section summarises observations and lessons learned in the implementation of the Simulink-based drivers and our experiments.

*Usability* Being able to manage these models in either the native tool or a model management framework requires metamodel understanding (model element types, their properties and operations). Model management programs should provide uniformity and predictability in how model elements are managed as part of the conciseness and expressiveness they offer compared to general-purpose languages. For example, in Epsilon CRUD operations on model element types share the same syntax regardless of the model’s underlying technology. This enables practitioners to focus on the model elements and the logic of their programs.

Uniformity can help to speed the learning process and make these programs easier to write and maintain. Section 3 evidences the multiple styles that MATLAB uses to manage different model elements types, within the same model, e.g. Simulink versus Stateflow, and between different model formats, e.g. Requirements versus Dictionary. It is not just the naming of the MATLAB functions that varies across operation types (e.g. setAttribute as property setter for requirement elements and set_param for Simulink elements), but also the arguments required by those functions. Similarly, different toolboxes use different notions of what constitutes an element id in their domain, e.g. Simulink sometimes uses the element id but most functions only work with their path property (their location) or their handle (a session based, non-persisted identifier). Furthermore, in the case of Simulink different parameters sometimes yield different result types, e.g. the find_system function can return handles or paths depending on whether the FindAll flag is active. A side-contribution of our approach is the unification of the syntax of several MATLAB toolboxes which can make it easier to focus on the core model management logic.

*Completeness* MATLAB and its Java API provide facilities to support the execution of CRUD operations on its model elements and the model itself. This API also provides an interface for a few MATLAB-specific data types such as structured arrays. In contrast, Simulink and dictionary models cannot be exported into any exchange format from this MATLAB, although more exporting facilities are available for Requirements including ReqIF. It is common that vendor tools are reticent to export their models into exchangeable data formats, e.g. to protect their intellectual property. However, when they do export them, sometimes they do so partially—like PTC with partial exports [49] and Simulink with ReqIF, which can make the round-trip engineering prohibitive (e.g. [49]) or complex (e.g. [29]).

**Table 5:** Performance improvement (%) by query and model

Query	Model 1	Model 2	Model 3	Model 4
1	97.52	98.95	99.43	99.76
2	55.03	56.15	50.20	55.09
3	91.80	97.20	98.82	99.48
4	69.92	87.68	94.95	98.01
5	91.73	93.95	93.71	93.88
6	96.36	98.11	98.07	96.75
7	97.58	98.72	98.64	97.33
8	98.78	99.45	99.46	99.10

In the case of Massif, the Simulink to EMF transformation is done by an external party. Among the disadvantages of this transformation is the lack of support for Stateflow elements and slightly different naming conventions to the ones used in MATLAB, different places to find element properties depending on the element type and the management of Simulink data types as strings. In contrast, model element types used in the Simulink EMC driver are closer to those managed by the MATLAB command line interface and also include Stateflow elements. In addition, by exploiting the MATLAB API facilities at runtime our Simulink EMC driver can also manipulate MATLAB specific data types.

*Performance* Several criteria can impact the performance of model management processes that involve Simulink models, e.g. the size of the model, the program complexity and the rate of model evolution. Our first experiment on large Simulink models showed that the cost of upfront Simulink-to-EMF transformation was particularly expensive in the Massif/EMF approach while the cost of the program execution was much lower than that of the Simulink EMC driver (by 2 orders of magnitude). In light of the program execution performance, the Massif/EMF seems convenient for large signed-off models (transformation cost paid once) that need to be extensively queried. In contrast, this same experiment showed that the overall execution process was reduced by up to 80% with the Simulink EMC driver, which concentrated the cost in the program execution. The overall execution performance makes the Simulink EMC driver better suited for continuously evolving models, otherwise recurrent transformations would be needed in Massif/EMF. We anticipated that the execution overhead in our approach was due to the cost of the MATLAB exchanges. Our proposed optimisations on operations on collections of Simulink model elements (Sect. 3.1.2) were able to reduce the number of MATLAB exchanges by not making them proportional to the collection size.

For smaller models, the decision of one approach or the other is more related to the model coverage offered by the approach and the relevance of the EMF model, i.e. its support in the model management tool and associated maintainability costs.

*Other* Model validation processes generally involve several iterations of checking constraints and fixing errors, unless the model is correct to start with. Similarly, model-to-model transformation and other model management programs may also result in the generation or modification of Simulink models. From our experiments, it is unclear the performance impact and completeness of the EMF-to-Simulink transformation although it is likely to have similar costs to the import procedure and similar issues to those found in other tools such as those mentioned for the ReqIF requirements imported by MATLAB [29] or the XMI models exported by PTC [49]. Our on-the-fly approach does not need to incur in round-trip engineering costs as it directly acts on the models themselves.

Our piecewise translation of model management constructs to MATLAB is convenient to deal with multiple (heterogeneous) models in the same model management program and to process the model information within the managing program. A complete translation of these constructs to a MATLAB program that executes just once would be more complex to orchestrate and to interact with from the model management program, e.g. to retrieve variable values that are assigned to elements from other models. The stark performance difference between the execution of MATLAB functions in Java or in its console suggests that further optimisations and strategies are required to reduce the number of exchanges with MATLAB and improve the performance of model management programs while still preserving their ability to interact with other models.

## Related work

It is often desirable to have a common framework to manage models from heterogeneous modelling technologies. Traceability tools such as Capra [23] and Yakindu [16] are examples of those frameworks, which need to be able to read models used at different stages of the development process in order to create and manage traces among their model elements. Other examples include model management frameworks such as Epsilon [19] and ATL [17], which offer a subset of task-specific languages for model navigation, validation, model-to-model or model-to-text transformations, etc., and which are able to interact with a number of models of arbitrary underlying technologies.

When model management frameworks do not offer support for a specific modelling technology such as Simulink, import and export facilities can be used to translate the models into a supported format. Possibly for reasons of protecting intellectual property, proprietary modelling tools do not always offer exporting facilities into open modelling formats such as XMI. MATLAB, in particular, does not offer any export or import facilities for Simulink models with other open-source modelling formats. To address this feature gap, the open-source Massif project led the development of import and export facilities between EMF and Simulink models. Massif internally uses MATLAB’s command line interface to parse the Simulink models and populate their EMF representation and vice versa.

The OSLC [34] is an initiative that aims to simplify the software tool integration problem among proprietary tools. Built atop the W3C Resource Description Framework (RDF), Linked Data and the REST architecture, OSLC provides a set of specifications targeted at different aspects of application and product life cycle management. OSLC is now being used by proprietary tool vendors (e.g. IBM Rational DOORS [15]) and some open source tools (e.g. [7]) who expose a range of services following these specifications. Nevertheless, the comprehensiveness of the information exposed by these services is at the discretion of the service provider. MATLAB does not officially provide an OSLC interface for its Simulink models, although the Eclipse Lyo [47] project provides an OSLC adaptor for Simulink [43] for MATLAB version R2013b, and Massif provides an OSLC adaptor for their EMF-compatible representations [14]. Reqtify [4] is a proprietary tool which exposes internal traceability information from Simulink models in a similar fashion to OSLC.

Transformations from SysML to Simulink models (and vice versa) have motivated several research works such as [3, 5, 32, 39]. [5, 32, 39] and [3] made use of model-to-text transformations with Acceleo [45] to produce MATLAB programs that on execution created the Simulink model. More specifically, [5, 32] generated several MATLAB scripts to populate different parts of the Simulink model, Chabibi et al. [3] proposed the use of a UML profile to annotate the SysML models before the MATLAB code generation, and [5, 39] suggested that to go back from Simulink to SysML the creation of a MATLAB script to parse Simulink models and produce an XML-based SysML model description file. In the domain of co-simulation, communicating between MATLAB Simulink and other frameworks is a common task. For example, Engel et al. [8] uses a software environment based on Ptolemy II [6] to run MATLAB scripts that get and set parameters of specific Simulink blocks and run simulations. As these works either use purposed SysML to Simulink transformations or focus on setting and getting parameter values of limited elements, they are not easily reusable for alternative model management scenarios such as querying the Simulink model or validating constraints. Examples of other works that used Simulink models external model management processes include [31] which performs independent translation of Simulink and Stateflow blocks into UPPAAL timed automata representations that are later combined and used in model checking and [11] which performs invariance checks on simplistic Simulink model representations written in JSON. In this regard, the Massif project and our approach facilitate the managing an EMF-compatible representation or the actual Simulink model (respectively) in a broader range of model management scenarios.

Our Simulink bridge built atop the Epsilon facilities is not the first one to bridge proprietary tools with the open-source model management languages of the Epsilon family. In [12], a spreadsheet driver was introduced to enable the manipulation of spreadsheets as models where element types were resolved from spreadsheet names, elements from rows and properties from columns while enabling flexible rules to resolve element references or change these conventions. Our approach is closer to that used by the PTC-IM driver presented in [49], where an interface with the PTC is used to manage the models. One difference with the PTC driver is that in MATLAB the API is not consistent and required commands to be built on demand. Additionally, MATLAB has a full-fledged language to manage its model elements that PTC does not, which allowed us to implement query optimisations. As in this work, one of the findings of [49] is that where performance is of essence, it is best to use the native tooling. In [49], the driver is evaluated against the native approach to manage the models by the tool, i.e. Visual Basic. In contrast, in this work our first experiment compares two different approaches to bridge Simulink models with model management frameworks, while the second experiment evaluates an approach to reduce the overhead of queries while also measuring the cost of communicating with MATLAB. A former driver for relational databases was proposed in [22] which generated SQL queries at runtime. The main difference between this approach and ours is the domain of application and non-uniform MATLAB API used to manage different model types. Kolovos et al. [22] investigate the use services provided by the underlying technology to optimise those provided at the proxy level in a similar fashion to what we do in this work although no evaluation is provided.

## Conclusions and future work

We have presented an approach to bridge Simulink models with model management frameworks that uses on-the-fly and on-demand translation of OCL-like statements into MATLAB commands. Given the widespread use of Simulink models in industry and the potentially large size of such models, our bridge offers an alternative approach to manage these without requiring their complete upfront transformation into an EMF-compatible representation therefore avoiding expensive transformation costs for large models and potential co-evolution procedures. Our public implementation, built atop Epsilon, enables comprehensive and uniform Simulink model, Simulink requirement and Simulink dictionary management, that includes Stateflow elements.

We have evaluated our implementation against a representative approach that requires an upfront model transformation into EMF set-up using facilities from the Massif project. This experiment measured the execution time of a model validation process evaluated on a sample of large publicly available Simulink models in GitHub (up to 1.141 MB and 9536 blocks) using both approaches. Our evaluation results support the claim that the transformation of large Simulink models into an EMF-compatible representation can be very expensive and shows that our bridge was able to reduce the overhead of this approach (mainly due to the transformation) by up to 80% in the validation process used in an experiment. Further evaluations showed that the cost of continuous MATLAB communication in our implementation is far from negligible which led us to introduce optimisations for operations that work on collections of Simulink and Stateflow model elements that were able to make these operations more efficient by up to 99%.

*Future Work* In light of the expensiveness of the communication with MATLAB through the Java API, in future work we will explore alternative mechanisms to reduce the impact of these communications which includes expanding our query optimisations to more complex queries. We are currently working on an approach that reduces the number of required exchanges with MATLAB by queueing the commands to be dispatched until the Epsilon program either finishes or has to use data computed in MATLAB. We would also like to add further support MATLAB Simulink-based models such as those provided by the Tests and Test Harness toolboxes. In addition, we plan to investigate alternatives to continue improving the efficiency of OCL expressions in large Simulink models and to add support for these optimisations to the Dictionary and Requirement drivers.

## Footnotes

1 The arrow on the left is not a transition.

2 The character “!” is used in Epsilon to separate the runtime name of the model from a model element *type* or *kind* available in that model.

3 Other collection operators such as select() and collect() are provided in EOL by default although drivers may override their behaviour.

4 Indexed data containers that can store any type of data.

5 Groups of data in containers that store any type of data.

6 Blocks that represent a reference to another model.

7 The use of the back-tick () is required when a type identifier contains spaces.

8 The only method that does not follow this structure is provided by the driver.

9 In the script, the model is identified at runtime as D.

10 We had access to one industry model that was 1.4 MB in size but for the experiment we had to find others in public repositories. To increase our chances to find complex models and to facilitate the collection procedure, we looked for models persisted in a file larger than 1 MB in size.

11 Inport, Outport, Goto, From and SubSystem are all subtypes of Block.

12 The temperature at which they start to heat.
